# Exploring ocean data: comprehensive approaches to data collection and the role of public databases in facilitating the interconnection between ocean and human health

**DOI:** 10.3389/fpubh.2026.1770936

**Published:** 2026-05-21

**Authors:** Anna Muratore, Lorenza Notargiacomo, Giorgia Mattei, Fulvio Ferrara

**Affiliations:** 1 National Center for Water Safety (CeNSiA), Istituto Superiore di Sanità, Rome, Italy; 2 Department of Chemistry, Sapienza University of Rome, Rome, Italy

**Keywords:** marine environmental monitoring, ocean and human health, ocean data, ocean database, ocean observation system, Ships of Opportunity

## Abstract

Since the year 2000, oceanic research has seen a surge in data collection, with approximately 500,000 sets of measurements for a single variable (e.g., temperature) recorded annually. Yet, further advancements are essential to deepen understanding of climate change phenomena, pollutant propagation, and the ocean-human health nexus. Analyzing Essential Ocean Variables (EOVs) designated by the Global Ocean Observing System (GOOS), such as temperature, salinity, pH, dissolved oxygen, phytoplankton distribution, coral cover and many others, is critical to assessing oceanic responses to anthropogenic pressures. Equally vital are persistent pollutants, antimicrobial resistance genes, human viruses, pathogenic bacteria, and micro- and nano-plastics, which underscore the inseparability of human and environmental health under an Ocean and Human Health framework. The public health sector can contribute and provide support for the continuous expansion of EOVs and this review seeks to contribute to this process, endorsing collaboration between public health experts and oceanographers to inform health policies that recognize oceans’ central role in human well-being. Over decades, a wide range of tools have been developed for the scientific community to measure EOVs, ranging from those requiring human intervention to automated systems. This narrative, not exhaustive review summarizes some of primary ocean observation technologies integrated within GOOS: satellites, drifting and moored buoys, research vessels and ships of opportunity (SOO), Animal Borne Ocean Sensors (AniBOS), and unmanned vehicles. Each one is examined in detail, highlighting characteristics and landmark projects like the Argo program and the FerryBox initiative, which have profoundly shaped oceanography. Accessing EOVs collected through these programs is fundamental to contextualize the new variables. Many databases are available to facilitate this process and key information will be provided. The review also explores emerging frontiers, particularly advancements in SOO through public-private collaborations. Framed from a public health perspective, it emphasizes the pivotal role of data collection and access in elucidating ocean-human health (OHH) interactions.

## 1 Introduction

The global ocean covers 71% of the Earth’s surface and holds approximately 97% of the Earth’s water. In addition, the ocean plays a key role in sequestering the 20–30% of the total anthropogenic CO_2_ emissions (1). The world’s oceans provide sustenance for billions of people, with 600 million livelihoods depending at least partially on fisheries and aquaculture (2). The oceans are crucial in setting climate, and in climate change. Their significance extends to the provision of water, food and overall wellbeing. It is noteworthy that they possess the capacity to adapt to changing conditions, even though the pressure exerted by humanity is unrelenting and progressively intensifying (3). While humanity continues to exert direct or indirect stressors on the oceans (4), it is also committed to studying the oceans themselves and how they respond to pressures. Indeed, the United Nations (UN) has declared the period 2021–2030 as the Decade of Ocean Science for Sustainable Development, known as the Ocean Decade (5). As outlined in the United Nations Sustainable Development Goals (SDG), Goal 13 is dedicated to the promotion of *Climate Action,* while Goal 14 concentrates on the preservation and conservation of marine ecosystems – *Life Below Water* (5). Throughout history, humankind has relied on the ocean for sustenance and other products, for early warning systems regarding impending danger (e.g., storms or invaders), for inspiration, beauty, commerce, and exploration. In the contemporary era, there has been a notably increase in the level of observation of the ocean (6).

In 2020 Brett et al., (7), introduced the concept of a “*data tsunami*,” a term that encapsulates the sheer volume of oceanic data collected since the early 1900s. This phenomenon has undergone a marked intensification since the year 2000, a period during which the annual number of casts exceeded 200,000, where a cast refers to a set of measurements for a single variable (e.g., temperature or salinity) at different depths. Since that time, there has been a stabilization at approximately 500,000 casts per year. These observations are collected using both autonomous and non-autonomous devices, with a significant portion of the efforts coordinated at a global scale by the Global Ocean Observing System (GOOS). Established in March 1991, the GOOS emerged in response to the calls from the Second World Climate Conference (Geneva 1990) and aims to enhance our comprehensive understanding and predictive capabilities concerning global climate and climate change, while acknowledging the paramount role of the oceans within the broader context of global climate system. The initiative was formalized by the United Nations Conference on Environment and Development (Rio de Janeiro, 1992) and the Intergovernmental Oceanographic Commission (IOC), with three main objectives: (1) to establish a coordinated international system for the collection of oceans and seas data, (2) to facilitate the processing of collected data for the purpose of generating prognostic environmental information services, (3) to stimulate research and development initiatives to enhance the efficacy of these services (8). GOOS has identified a set of Essential Ocean Variables (EOVs) that must be monitored. These include sea surface temperature, salinity, sea level, sea ice, ocean color for biological activity; subsurface temperatures, salinities, currents, nutrients, carbon dioxide concentrations, phytoplankton distribution, coral and mangrove cover and many others (9). To monitor these variables, a combination of satellite and *in situ* measurements is employed. Nowadays there are 13 members into the GOOS network that provide long-term, global, high-quality, in-situ ocean observations (10): Argo (11–13), Data Buoy Cooperation Panel (DBCP) (14), Ship Observation Team (SOT) (15), Automated Shipboard Aerological Programme (ASAP), Voluntary Observing Ships (VOS), Ships of Opportunity Programme (SOOP), Global Sea Level Observing System (GSLOS) (16), Animal Borne Ocean Sensors (AniBOS) (17, 18), Global Ocean Ship-Based Hydrographic Investigations Programme (GO-SHIP), OceanSITES, The Global High Frequency Radar Network (19), Ocean Gliders (20, 21) and Tsunami Buoys (Deep-ocean Assessment Reporting of Tsunamis, DART) (22). The observations from these sources are accessible via OceanOPS platform (23) and in late 2025 GOOS published an updated Status Report, available online (24), reporting 120,000 ocean observations per day and 9,389 ocean observing platforms. The following sections will delve into some of the aforementioned programs, offering a more thorough examination. However, it is important to acknowledge that numerous programs outside the GOOS network contribute to the topics discussed herein. These additional programs will not be explored in this review.

The *tsunami wave of data* is stable, but there is a need for further different types of data to build one of the objectives of this decade: the Digital Twins of the Ocean (DTO). DTO are numerical computed simulated models that create high-fidelity digital replicas of the global ocean, assimilating real-time observations for model calibration (data assimilation) and verification (validation). Since 2021 the European Commission has allocated approximately €15 million annually, through the Mission Restore our Ocean and Waters work program, with the objective of developing the European Digital Twin Ocean (25), which aims to create a comprehensive digital replica of the global marine environment, integrating all the variables that can serve as crucial tools for decision-makers. In order to enhance both the quantity and the quality of observations, two primary strategies can be pursued: the first involves the expansion of the suite of instruments to measure a greater number of properties over a more extensive range of scales, and the second involves the deployment of additional instruments to cover larger areas over extended periods (26).

DTO can be utilized for the purpose of marine pollution modelling and prediction, as well as in efforts to mitigate and prevent overfishing, all elements fundamental for public health research as detailed in Section 2. Nonetheless, significant economic and technological barriers persist (27). The existing body of data regarding fundamental oceanic processes and phenomena is incomplete, owing to the persistent gaps in scientific knowledge and technological development. The global ocean is vast, and satellite technology currently available has a resolution of 100 km or more (27). A notable breakthrough occurred in December 2022 with the launch of the Surface Water and Ocean Topography (SWOT) satellite, a collaborative project between NASA and the Centre National d’Etudes Spatiales (CNES) (28, 29). The objective of this satellite is to achieve a resolution of approximately 20 km, though a resolution of around 1 km remains a formidable challenge (27). The Argo Program, along with other profiling floats, has been unable to adequately address the dearth of data. Presently, March 2026, there are approximately 4,300 Argo profiling floats (30) and other 1,500 surface drifters in the oceans, providing observations with a resolution of 200–300 km and 400–500 km, respectively, (27). Moreover, substantial technological challenges pertain to data compatibility, processing, and dissemination. The sheer volume of observations is substantial, yet it is dispersed across numerous disparate platforms that often lack standardized formats. This has created challenges in harmonization and interoperability (27). However, it is also possible to obtain useful information by starting with an understanding of the available observations collection platforms and the available databases described in this review.

Over the years it has become essential to recognize that massive data collection is fundamental to investigate the escalating magnitude of anthropogenic pressures on the marine environment. That pressure include both climatic stressors as CO_2_ emission leading to ocean warming, acidification and sea level rise, and non-climatic stressors as plastic pollution, industrial contaminants and oil spills, which are linked to agriculture and urban development (4). The human activities have the potential to adversely impact environmental health, thereby raising concerns for human health as well. The presence of pollutants in the food chain has contributed to a decline in biodiversity, adversely affecting the quality and availability of seafood (31, 32). The progression of climate change and the growing impact of human activities on the ecosystem have prompted oceanography and all other relevant disciplines to delve even deeper into our understanding of this crucial phenomenon.

In response to the interconnected issues that have emerged, a novel discipline, termed Ocean and Human Health (OHH) has been proposed (33, 34). This emerging field encourages collaboration between public health and oceanography and this review aims to address this need, from the perspective of Public Health and Planetary Health (31), viewing human and environmental health as inseparably linked within the OHH scenario. In addition to currents EOVs, new variables related to public health can be considered and studied within the oceanic environment. Notable examples include Persistent Organic Pollutants (POPs) (35), microplastics (36), and Antibiotic Resistance Genes (ARGs) present in the ocean environment (37). It is therefore essential to use EOVs to contextualize these new variables and to access this information through public databases.

In conclusion, this narrative review aims to provide a broad, though not exhaustive, overview and context, allowing for the synthesis of diverse studies. It will present the Ocean and Human Health approach (Section 2) and the data collection methodologies employed by oceanographers (Section 3), along with the available databases (Section 4). This review will also highlight the need for new variables that can be measured in a OHH scenario (e.g., POPs concentration), using not only research vessels, but also through new frontiers in ships of opportunity, discussed in Section 5. The review was conducted by selecting peer-reviewed literature, without a specific time frame but prioritizing recent publications, through various platforms, such as Scopus and Google Scholar and using specific keywords related to various data acquisition methods, such as “AniBos,” “profiling AND floats,” “environmental AND satellites,” “ocean AND data,” and many others. The selection of results was based on the inclusion of the most relevant publications. Another proportion of the research was conducted by accessing on official websites of GOOS-affiliated programs, as well as other relevant programs, in order to consult the most up-to-date data regarding active data collection platforms. A meta-analysis was not conducted; rather, a thematic synthesis was employed to facilitate the discussion regarding OHH, new variables, and SOO.

## 2 A brief excursus into the ocean and human health: a compendium of metrics for assessment

The relationship between humankind and the world’s oceans is a long-standing one, with an interaction that has spanned millennia. Since the early 2000s, there has been a marked increase in research in this field, driven by growing awareness of the detrimental impacts of pollution, which initially affected the atmosphere and more recently reached the oceans. The OHH discipline (38) has given rise to *ad hoc* projects, and integrates the principles of One Health (39), and Planetary Health (31) by recognizing that the health of oceans, humans, and the environment are interconnected and mutually dependent. One Health emphasizes biological interactions between the health of humans, animals and ecosystems, while Planetary Health is a solutions-oriented, transdisciplinary field, focused on analyzing the impacts of human disruptions to Earth’s natural systems on human health (40). The relationship between ocean health and human health is intricately intertwined with the historical development of civilization. This relationship is characterized by an escalating demand for water and energy, advancements in agriculture and industry, and most notably, the substantial release of carbon dioxide emissions into the atmosphere. The increase in atmospheric carbon dioxide levels has been identified as the primary catalyst for a series of interconnected events, including ocean warming, ocean acidification, and the intensification of extreme weather phenomena, which have been shown to lead to an increase in pollutants handling. These events, in turn, contribute to sea level rise, deoxygenation, biodiversity loss, and the dissemination of microbiological species (e.g., viruses and *Vibrio*) to different latitudes. The consequences for human health are manifold, encompassing the degradation of coastal environments, the decline in fish stocks, the spread of diseases, migration, malnutrition, and conflicts over resources (31). In addition to climatic stressors, which are recognized as being indirectly related to human activity, direct stressors on marine environments must be considered (4). Plastic pollution, for instance, has been demonstrated to lead to microplastic dispersal, which can also act as a vector for pathogens (41). Nutrient spillage, for example nitrogen and phosphorus, has been demonstrated to induce the formation of harmful algal blooms (HABs), which are known to produce cyanotoxins that have been shown to be hepatotoxic, neurotoxic, or cytotoxic (42–44). Furthermore, spillage of POPs (35) is a matter of concern, as the accumulation of POPs in the food chain can exert a deleterious effect on human health, manifesting in a variety of ways ranging from cardiovascular diseases to neurological and developmental effects, and from metabolic diseases to cancer (33). These pollutants are distinguished by their high persistence in the environment, bioaccumulation, and biomagnification within the trophic chain. Among POPs, Polychlorinated Biphenyls (PCBs), together with methylmercury, are the ocean pollutants whose human health effects are best understood, in fact exist evidences that exposure of infants in utero through maternal consumption of contaminated seafood can damage developing brains. Chemicals such phthalates, bisphenol A, flame retardants and perfluorinated chemicals, released into the sea also from plastic waste, can act as endocrine disruptors and damage nervous system (33). In addition, pharmaceutical pollutants have the potential to induce the emergence of Antimicrobial Resistance Bacteria (ARBs) and select ARGs, observed in remote oceanic regions as well (45). Additional pollutants of concern include oil spills (46) and heavy metal spills (47), as well as the introduction of allochthonous bacteria, viral species and transportation and diffusion of these pollutants via vessel ballast water (48).

The diffusion mechanism of POPs and other contaminants depends on the chemical and physical characteristics and origins (inland or maritime). The sources of contamination may include urban areas, agricultural runoff, or industrial or urban wastewater. These pollutants traverse long distances through the atmosphere (they may undergo wet and dry deposition or volatilization phenomena) or enter the seas horizontally, propelled by oceanic currents generated by winds and Earth’s rotation, or vertically through physical upwelling, the direction of which is influenced by differences in temperature and salinity. Additionally, biological pumping, driven by the affinity of pollutants to organic matter or ingestion by organisms, also aids dispersal (49, 50). The vertical movement of water masses can also be attributed to upwelling, where winds perpendicular to the coastline displace heated surface water, causing the underlying cold, nutrient-rich deep water to rise to the surface. Sediments represent a significant source of seawater pollution, due to the presence of molecules with a high affinity for organic matter. These molecules can be adsorbed and resuspended, and the soil itself can act as a reservoir for this pollution. The chemical properties of these pollutants, including their affinity for organic matter, govern their environmental fate and transport mechanisms: redox charges regulate the transformation of chemicals by microbes, lipophilicity dictates the bioaccumulation potential in the adipose tissue. These molecular properties are pivotal in determining the metabolic degradation pathways of compounds within an organism preventing the molecule from accumulating in the tissue during the organism’s lifetime, resulting in higher concentrations in older individuals within a population compared to younger ones. Another notable biotic phenomenon is biomagnification along the trophic levels, due to the consumption of large quantities of fish by predators at the upper levels of the trophic chain, resulting in concentrations that increase at the successive trophic level and which exceed the concentrations observed in the surrounding water (51).

As already mentioned, temperature and salinity regulate the vertical movement of water masses and, consequently, can have an important role in the dispersion of contaminants. Therefore, access to these variables can be useful for analyzing pollutant concentration data. These variables, along with chlorophyll, can also be utilized for contextualizing microbiological data (52).

From the above, it is evident that the well-being of the ocean is inextricably linked to that of human health. The preservation of oceanic ecosystems is imperative for two fundamental reasons. First, these ecosystems serve as a vital reservoir of water and oxygen, which is crucial for sustaining life on Earth. Second, they play a pivotal function in regulating climate. The ocean plays a substantial role in global trade, accounting for 76% of international cargo transport. Moreover, it functions as a substantial catalyst for economic growth by leveraging mineral resources, facilitating renewable energy production, and promoting the use of natural products and biotechnological applications. The ocean also provides a substantial source of sustenance, with a recorded global capture fisheries production of 92.3 million tons in 2022 (53), and serves as a valuable source of ingredients for the pharmaceutical industry. Furthermore, oceans are recognized as significant sources of aesthetic value, offering opportunities for mental well-being and recreational activities (54).

A comprehensive analysis of this intricate scenario, reveals that, in addition to EOVs, several other variables could be monitored to obtain a comprehensive understanding of ocean health. These include the concentration of POPs and heavy metals, the distribution of microbiological and virological facies, and the distribution of microplastics. These new variables would require sophisticated equipment and advanced laboratory analysis on seawater to achieve concentrations as low as part per trillion (ppt) as in the case of POPs. The primary challenge in investigating chemical and microbiological variables is the sampling of remote ocean regions, as opposed to coastal environments alone. This subject will be addressed in Section 3.5, which discusses research vessels and new frontiers in ships of opportunity.

## 3 Methods of data collection

To comprehend the influence of climate change and human activities on environment and human health, it is necessary to monitor and study the oceans in conjunction with other systems, including the atmosphere, biosphere, cryosphere and geosphere, while to understand how the oceans are reacting to anthropogenic pressures, it is necessary to consider several components that include (a) routine real-time observations, (b) numerical models, (c) data assimilation techniques and (d) the ability to disseminate the products to stakeholders (6).

Essential ocean variables can be measured through two distinct categories: *in situ* methods and remote sensing. Despite the logistical, financial and scaling difficulties, *in situ* methods (e.g., oceanographic buoys, animals with sensors, autonomous vehicles and research vessels or ships of opportunity) are vastly superior compared to remote sensing methods when dealing with localized measurements, allowing for maximum accuracy and higher spatial and/or temporal resolution. However, when global coverage is required, remote sensing or satellite observations must be implemented, but sensors mounted on satellites have limitations, primarily related to low resolution, atmospheric attenuation in certain parts of the electromagnetic spectrum and last but not least measurements limited to ocean surface (55). In a multitude of scenarios, the two methodologies prove to be complementary. Satellite observation provides a comprehensive global perspective, while in situ measurements offer a detailed mesoscale (horizontal scale less than 100 km) view. These measurements are essential for augmenting satellite observations, particularly in subsurface regions, and for ensuring the accuracy and reliability (6, 56).

This section is intended for readers who are interested in learning more about how some of the EOVs are measured and who may not be familiar with all the details presented. As previously mentioned, EOVs are fundamental to analyze and contextualize new variables of public health interest (e.g., POPs concentration or ARGs presence) data. The section provides an overview of the available information selected by the authors. The following sections offer an introduction to the sensors, their basic operation, the networks and their development over time, and the three categories in which oceanographic instruments can be classified: Lagrangian platforms, which passively follow the water; Eulerian platforms, which remain nearly stationary; and self-propelled platforms, which include ships and gliders (57). All are further outlined in Table 1.

**Table 1:** Overview of main characteristics and limitantions of type of instruments.

**Methodology**	**Type of instrument**	**Main characteristics**	**Limitations**
Remote sensing	Environmental Satellites	Autonomous Nearly global coverage Long operational life	Low resolution and influenced by weather condition High cost
In situ	Surface drifters and profiling floats	Autonomous Small dimension Drift with currents and winds for surface drifters. Profiling floats are less impacted by winds Profiling floats capable of reaching deep open ocean and remote regions Low individual unit cost	Limited operational life at the end of which they become waste Instruments can only be calibrated pre-deployment, but post deployment validation can be done
	Moorings	Autonomous Capable of surface and sub-surface measurement Big dimensions compared to surface drifters or profiling floats.Long operational life Capable of mounting many sensors also for tsunami alert and atmospheric monitoring Instruments can be routinely calibrated so generate high quality data	Maintenance costs Influenced by weather conditions
	Animals with sensors	Autonomous Capable of reaching remote unsampled regions	Animal discomfort Low quality data Transmission opportunities brief, infrequent and generally not controllable
	Gliders	Small dimension Capable of reaching deep ocean Recoverable and reusable	Logistic costs for deployments and recovery Specialized personnel required Hard time maintaining their course Problems flying perpendicular across to strong currents
	Research vessels	Designed for oceanography research Capable of collecting seawater and performing multiple analysis in addition to EOVs	Limit spatial and time coverage Specialized personnel required
	Ships of opportunity	Can take advantage of existing and repeated routes Capable of collecting seawater and performing multiple analysis including EOVs and numerous different atmospheric sensors Reduced cost (compared to research vessel) Possibility of automatic sampling	Not design for oceanographic research Unexpected route changes Limit spatial and time coverage Instrumentation and ship to shore data communication system maintenance

### 3.1 Environmental satellite systems

Environmental satellites are defined as constellations of multiple instruments that are placed into orbit over an extended period, to monitor oceanic and atmospheric parameters, to provide weather forecasts and to facilitate climate monitoring. Satellites offer long-term, continuous, global, high-space-and-time-resolution observations for key ocean parameters, including sea level and ocean circulation, sea surface temperature, ocean color, sea ice, waves and winds (56).

The inaugural satellite dedicated to ocean observation was launched in 1997, with the *Seasat* satellite being capable of measuring Sea Surface Height (SSH), which can be used to estimate surface and subsurface geostrophic circulation. Two other fundamental variables are Sea Surface Temperature (SST) and Sea Surface Salinity (SSS), which are utilized to compute surface density. Density gradients in turn, contribute to surface currents, water mass formation, mixed layer depth, and so forth. Velocity and ocean currents can also be reconstructed from SSH, surface winds and SST within a physical model. The first measurements of SST were taken in the early 1980s using the Advanced Very High-Resolution Radiometer (AVHRR), while SSS measurements only became available in 2009 with the Soil Moisture and Ocean Salinity satellite (6). Since these initial advancements, both Europe and the USA have invested significantly in the development of satellite constellations for environmental monitoring purposes.

The European program *Copernicus* and the European Operational Satellite Agency (ENUMESAT) are responsible for the management of the *Sentinel* network (58), a constellation of 7 satellites, orbiting at an altitude of 700 to 800 km (Low Earth Orbit, LEO). The Sentinel-3A and-3B satellites are dedicated to the observation of ocean color, while the Sentinel-6A satellite is tasked with the measurement of global sea surface height and temperature changes in the troposphere and stratosphere. The launch of the second Sentinel 6, designated 6B, has just been launched in November 2025. In the United States, NASA and other partners have launched the SWOT satellite, the first satellite capable of measuring SSH with 15–30 km resolution (29).

The fundamental principle underlying remote sensing is the detection of electromagnetic signals. The specific target variable dictates the range of the electromagnetic spectrum that is considered, with the measurement method being either active or passive. Active measurements entail the emission of a signal (in the microwave) and the subsequent detection of the reflected signal. A case in point is SSH, where the travel time and strength of the signals are taken into consideration. Conversely, passive measurements do not involve emission, but rather the detection of naturally emitted and/or reflected signals (sunlight) from the sea. Signals from different parts of the electromagnetic spectrum can be utilized, including the visible light (400–700 nm) for ocean color, to measure chlorophyll-a, suspended particulate matter, organic and inorganic carbon, and infrared (0.7–20 μm) for SST. It is also possible to measure these parameters in the microwave (1–30 cm) during cloudy conditions (56). The primary concern with satellite measurement is the fact that not all parts of the spectrum possess equivalent penetrative properties. Atmospheric transparency to microwave radiation is evident, whereas visible and infrared light can be attenuated by clouds, resulting in missing values. However, recent technological advancements have enabled the reconstruction of these data using various techniques, thus facilitating their availability for research purposes (55). In both active and passive scenarios, it is imperative to consider the atmospheric propagation of the signal and the atmospheric emission of the signal when attempting to extract the sea surface signal (56). In order to provide a more detailed overview, it is possible to equip each satellite with a number of different types of instruments: a spectroradiometer, a light measurement tool that measures sunlight reflected by oceans from visible to medium-infrared, an infrared radiometer, tools for measuring radiation reflected by surfaces for temperature estimation, a microwave radiometer, that measures the radiation emitted as microwave for atmospheric parameters but also SST and SSS, satellite altimeters, which make a global measurement of the surface topography by measuring how long it takes for a short electromagnetic pulse sent from the satellite to reach the surface and come back after being reflected to measure SSH, satellite scatterometer, a radar sensor to measure the scattering effect, and synthetic-aperture radar (SAR), a radar that can be used to create two or three dimensions images of the Earth surface (59).

The primary feature of remote sensing is undoubtedly its capacity for global coverage, enabling the observation of the entire surface, a feat that would be unattainable through different kind of *in situ* measurements. Satellites measure ocean surface, including radiation, and thus complement systems such as buoys and gliders that are able to explore the deep ocean.

### 3.2 Oceanographic buoys

A significant proportion of the buoys that are utilized for the purpose of oceanic observation are components of an international network, the funding, management and maintenance of which is the responsibility of a variety of national and international institutions. These institutions provide guidelines and best practices. Collaboration is a fundamental aspect of the operation of buoys, particularly those that are moored. These buoys necessitate the execution of substantial fieldwork, due to the harsh environment of the ocean, the potential damage to instruments, the risk of data loss, the possibility of battery or anchor cable failure, and the detrimental effects of salt, dust, and biofouling on sensors (60).

Oceanographic buoys are instruments designed to collect data on specific variables, depending on the type of sensors installed and transmit the information to a satellite network. In this capacity, satellite act as a conduit between the buoy and the shore, where the data are processed. There are different types of buoys that can be used for short-term process studies or as part of long-term programs. These include anchored or drifting buoys, surface or subsurface buoys. Surface buoys have the capacity to be anchored to the seafloor or to move following the currents. Measurements of the deep water can be performed with stationary buoys that have sensors at specific depths along the rope that anchors them to the seafloor or through devices capable of moving three dimensionally through the water column, collecting measurements as the buoy ascends or descends (60), as the Argo profiling floats. A plethora of parameters can be measured, ranging from temperature and seawater salinity to wind speed and direction, as well as biogeochemical parameters such as the Biogeochemical-Argo (BGC-Argo) (61). As previously mentioned, data transmission on shore is achieved through satellite and data centers apply automated quality control procedures and make the information available to the public. Thereafter, they perform a reanalysis to produce delayed-mode versions of the data sets, with a higher quality (60).

#### 3.2.1 Surface drifters and profiling floats

Oceanographic floats are designed to move with ocean currents and can be focused on surface measurements alone (surface drifters) or alternatively, they can collect casts along the water column (profiling floats) up to 2000 m depth. Prior to 2017 the majority of drifters, including those employed in the Global Drifter Program (GDP) (62), utilized the Argos satellite tracking system (63), transmit information every hour, offering a location accuracy of 400–450 m, after 2017, more than 98% of drifters acquire location with GPS with an accuracy of few meters using the Iridium satellite constellation (60, 64). This trend has also been adopted by the Argo Program which now, March 2026, has over 4,300 floats in operation. Of these, only less than 2% continue to use the Argos system (see data available and constantly updated at (30)). The advent of technology in this scenario proved to be a pivotal moment, particularly given that the trajectory of the early drifters was entrusted to a system analogous to that of bottled messages, whereby contact was referred to by communicating the date and location of the find. The trajectory was then calculated only from the point of start to the point of end (57). Historically the first drifter to be deployed is attributed Theophrastus who hypothesized that the Atlantic Ocean flows into the Mediterranean Sea and used a bottle to test this theory (57).

##### 3.2.1.1 Surface drifters

**Figure 1:**
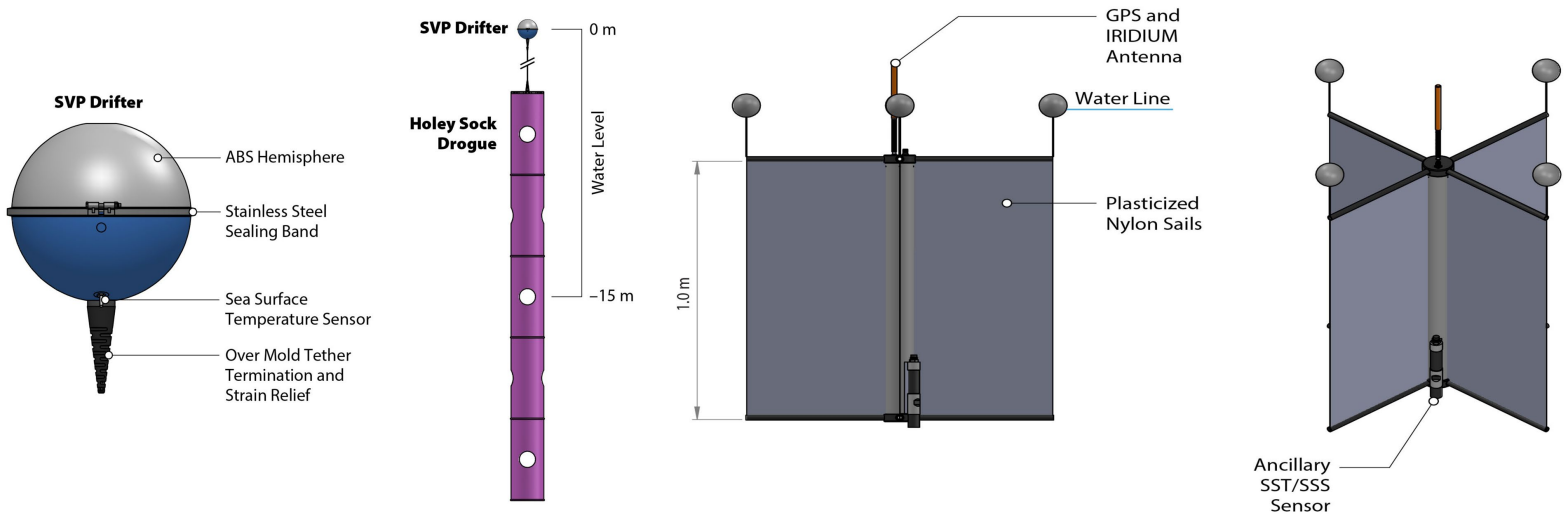
SVP drifter (left) and CODE drifter (right), from https://gdp.ucsd.edu/ldl/svp/.

The advent of modern surface drifters can be traced to the early 1980s, characterized by their low cost, lightweight nature, and ease of deployment. These buoys were utilized as part of the Tropical Ocean Global Atmosphere experiment (1985–1994) (65) and the World Ocean Circulation Experiment (1990–2002) (59, 66, 67). Several designed were created: the Surface Velocity Program Drifter (SVP) (Figure 1) (68), the Coastal Ocean Dynamics Experiment Drifter (CODE) and the Lagrangian Submesoscale Experiment Drifters. The SVP, composed of a sea anchor at a depth of 15 m, is one of the most widely used by the GDP. It follows mixed layer currents and is attached by a tether to a surface float containing the electronics, sensors, batteries, and the transmitter (60). The sea surface sensor, which is positioned just beneath the sphere, and a miniaturized version, were introduced in 1992. These sensors are characterized by their reduced size and weight and have an average operational life of approximately 450 days (57). The second model, the CODE (Figure 1), consists of a vertical tube containing GPS and Iridium antennae, with four vanes attached to 50-cm-long arms that form a cruciform drogue, at the end of each arm a surface floats is attached (57).

The third and final model of drifters under discussion was introduced in the aftermath of the Deepwater Horizon oil spill in the Gulf of Mexico in 2010. The impetus for this model was the necessity for compact, lightweight, rapidly deployable, and mass-producible drifters that were biodegradable to the greatest extent possible in order to minimize long-term damage to the environment (57). The final design consists of a floating torus measuring 0.35 m in diameter, equipped with a GPS unit, batteries, and a drogue comprising two interlocking square pieces. Fabricated from bioplastic, the drifter can survive in the ocean for a period of 4–6 months. The drifter was designed for mesoscale analysis, and 1,000 were deployed in the Gulf of Mexico during the winter of 2016 (57).

The coordination of buoys programs is currently undertaken by the Data Buoys Coordination Panel (DBCP), a constituent element of the GOOS. Formed in 1985, the DBCP is responsible for the coordination of the drifting and moored buoy components of a number of significant sub-programs (14).

**Figure 2:**
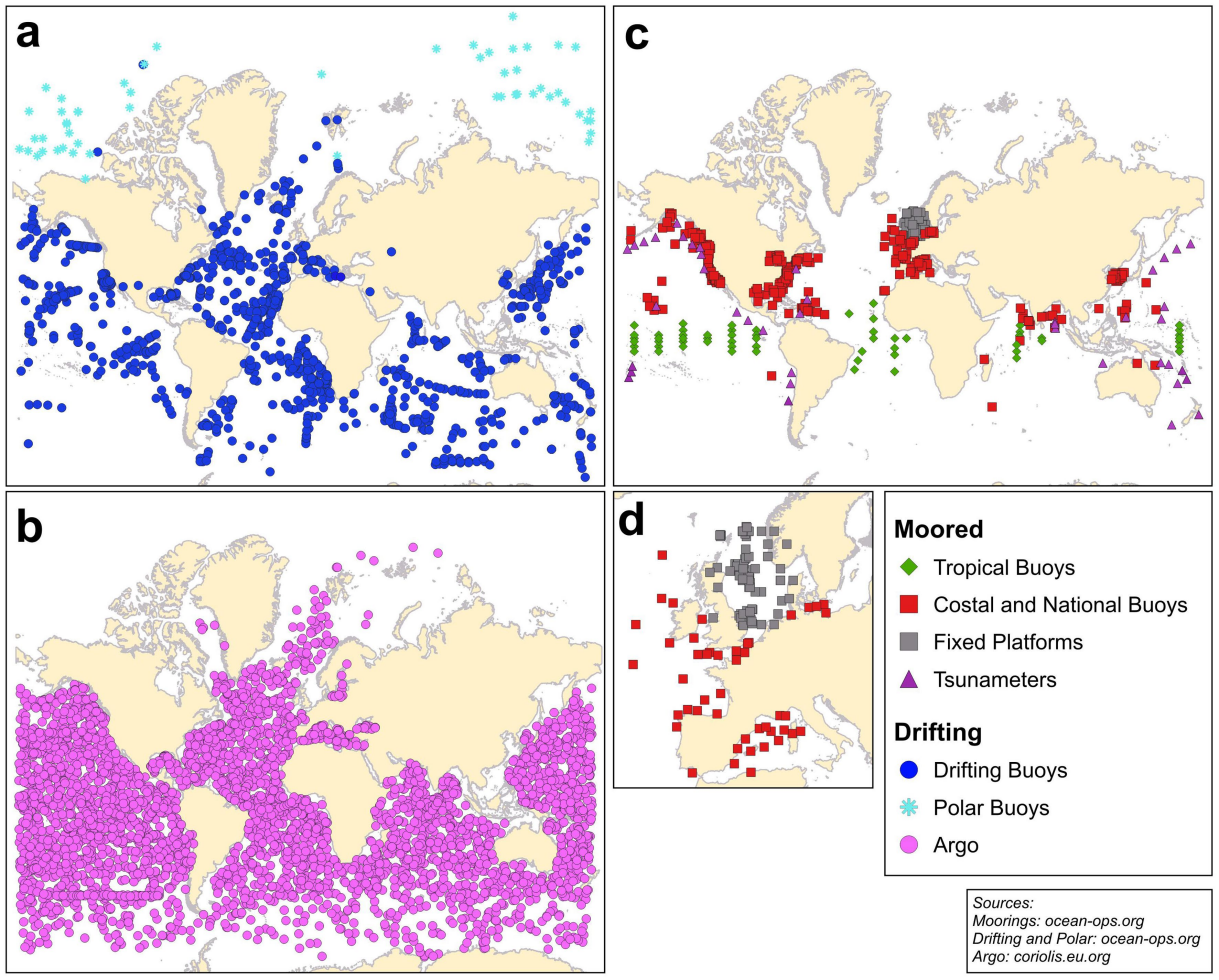
Maps reporting ocenographic buoys. **(a)** Surface drifters, **(b)** Argo profiling floats, **(c)** Moorings,with a zoom on Europe **(d)**. Data about mooring and surface drifters are downloaded from OceanOPS (https://www.ocean-ops.org/board#), data about Argo profiling floats comes from coriolis.eu.org.

Accessing the data available in the OceanOPS portal (see *Available databases* for further details) reveals that there are currently more than 1,200 drifting buoys registered (Figure 2a). A subset of these is classified as polar or ice buoys due to their construction for extreme climate conditions, with electronics and lithium batteries capable of operating at temperatures as low as −50 °C.

##### 3.2.1.2 Profiling floats

One of the most well-known profiling floats program is the Argo Program, whose name is inspired by the ship of Jason and his crew, the Argonauts (13). As previously mentioned in the introduction, this paradigm shift represents a major advance in the study of the world’s oceans, particularly in regard to the deep ocean. Prior to the implementation of the Argo Program in 1999, in situ observations were predominantly collected from ships, moorings, or surface drifters, generally in the Northern hemisphere, with a significant proportion of the world’s oceans remaining unsampled (13). Upon establishment, the program’s objective was to deploy 3,000 floats by 2007, a target that was met in November of that year (12) The current total stands at approximately 4,300 floats (30) (Figure 2b), positioning Argo as a prominent element of the GOOS. Nowadays the Program has a new design called *OneArgo*, with the ambition of achieving global coverage, full depth and measuring biogeochemical variables (69).

**Figure 3:**
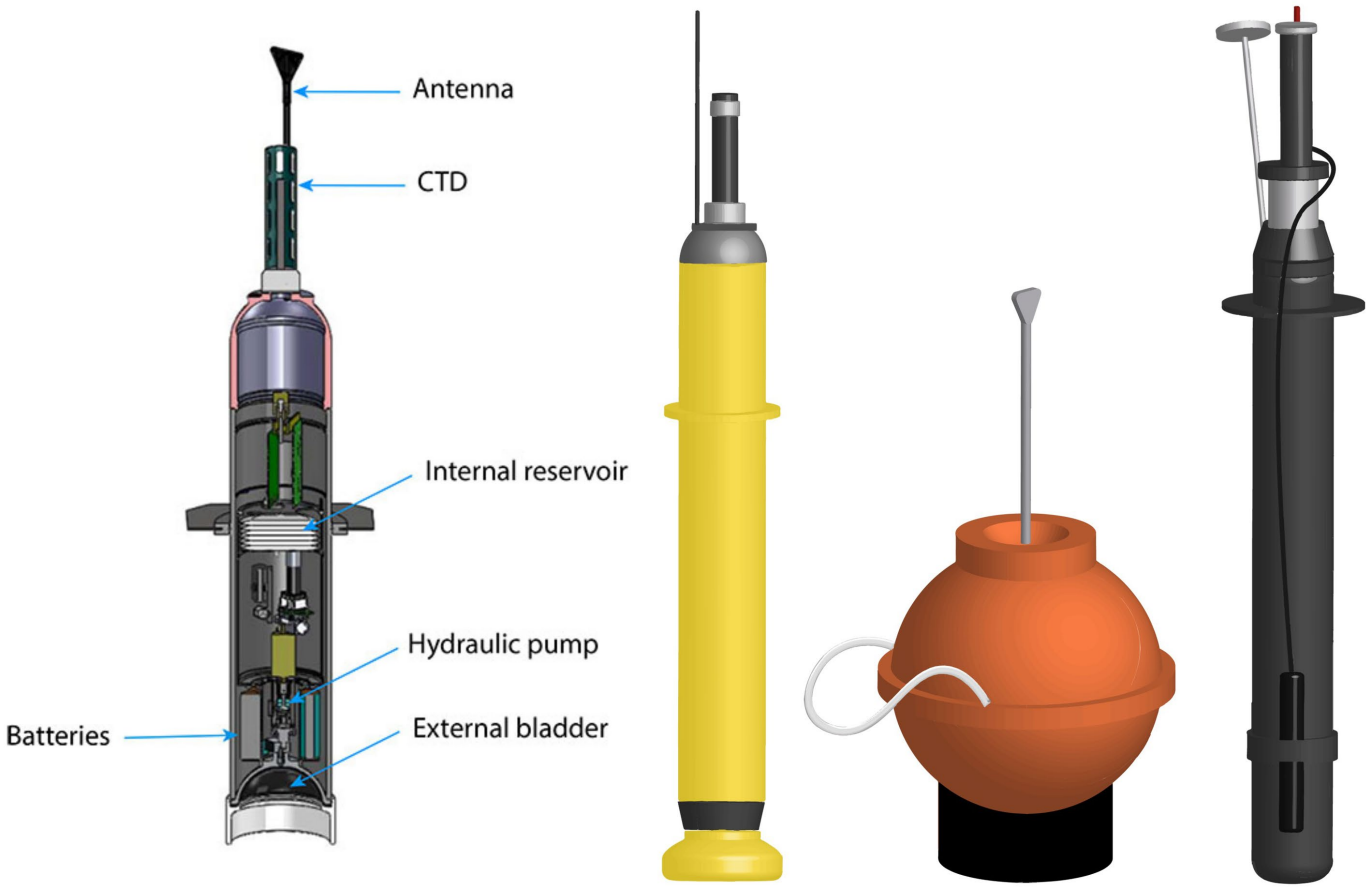
Internal structure and types of Argo floats from: https://argo.ucsd.edu. From left to right: internal structure of the Core Argo float, Core Argo float, 6,000 m Deep Argo float, and BGC-Argo.

**Figure 4:**
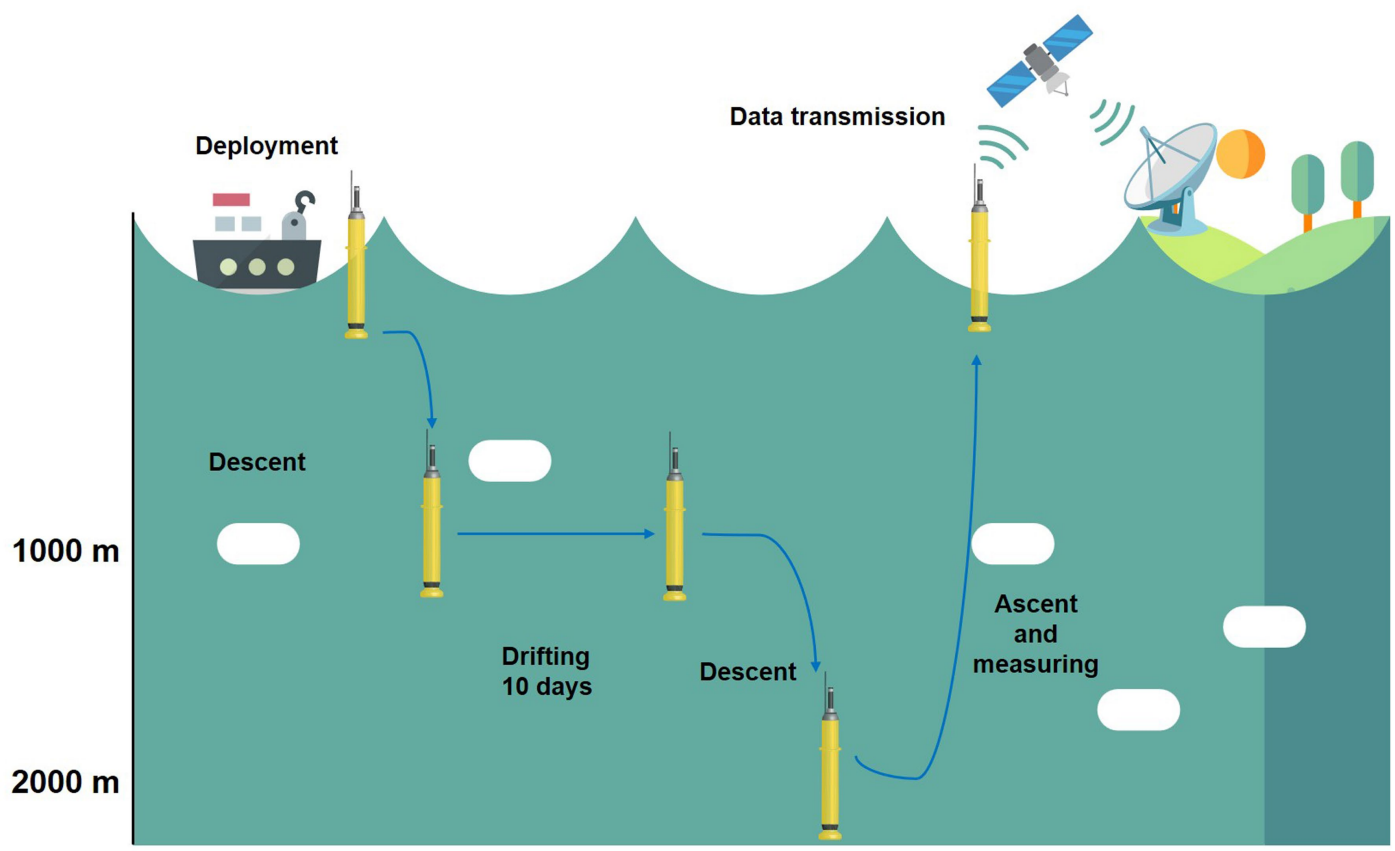
Cycle of an Argo floats.

Argo floats are 2 m long robotic devices, equipped with a pumping system capable of adjusting depth by changing the buoyancy (12). The upper part of the floats contains the antennas, the CTD (Conductivity, Temperature and Depth) sensors, where conductivity provides an estimate of salinity, and any additional sensors that may be installed, depending on the model and the purpose of the float. The internal configuration of the buoy typically assumes a cylindrical form, housing the electronic components that regulate the sensor functions and the data acquisition process. Additionally, the hydraulic pumping system and the lithium batteries are integrated within the buoy’s body (Figure 3) (70). The operational life of a buoy is approximately 10 days, following which it reaches a depth of 1,000 m (referred to as the ‘parking depth’). Thereafter, it drifts for approximately 10 days before descending to a depth of between 2000 or in the case of Deep Argo 6,000 m. During the subsequent ascent process, all the observations are collected, stored inside the local memory and then transmitted to the satellite system when the float reaches the surface (Figure 4). The cycle begins again for a mean life of 5 years, after which the lithium batteries run out of charge. As previously mentioned, the transmission of the majority of data (97%) is facilitated by the Iridium satellite network, a pivotal element of the program. This enables the transmission of all observations in less than 20 min, thereby enabling the probe to immediately initiate a new cycle. Additionally, the network facilitates two-way communication, allowing for the transmission of instructions to the float for troubleshooting or mission modification (13). The subsequent processing of these information onshore ensures that they meet the requisite standards, with accuracies of 0.005 °C and 0.01 salinity units, along with a pressure accuracy of 2.5 dbars. Experience has demonstrated that approximately 80% of the raw profile transmitted from the floats meets these standards, with minimal or no correction required. The remaining 20% of the data undergoes correction through the utilization of delayed-mode quality control procedures. Presently, approximately 90% of Argo profiles are distributed electronically within 24 h of acquisition (12). In planforms as EuroArgo (30) is possible to visualize and download the data (in good or bad quality) for buoy and for each cycle.

The Iridium satellite network has been demonstrated to facilitate enhanced geographical coverage of the sea ice zones in the Southern and Arctic Oceans. This advancement is attributed also to the refinement of the float algorithm, which has been developed to circumvent the potential obstruction of the ascent by ice packs. The float algorithm is predicated on the hypothesis that the presence of sea ice is associated with the temperature of the water column. Consequently, this enhancement has enabled the acquisition of measurements in these regions of paramount importance. In summary, the float measures the water column temperature, and the algorithm calculates the median temperature between two depths during ascent. If the median temperature is less than a specified threshold, the presence of sea ice is assumed, and the float aborts its ascent, storing the profile on board and descending again, thus initiating the next cycle. With the Iridium satellite, floats are capable of collecting and storing information during winter and transmitting all of them during early summer (13).

##### 3.2.1.3 Deep and biogeochemical Argo

In addition to the conventional buoys that attain a maximum depth of 2000 m, designated as Core Argo, two supplementary networks have been implemented: the Deep Argo, a modified version of the buoy capable of reaching depths of up to 6,000 m, and the Biogeochemical Argo (BGC) floats, which are equipped with optical and oxygen sensors. Despite the fact that the deep ocean, i.e., the layer extending below 2000 m, constitutes 50% of the total volume of the ocean, historical floats profiles extend only 10% below 2000 m. The Deep Argo therefore aims to improve knowledge of a fundamental part of the ocean environment. Presently, approximately 200 Deep Argo floats, with the capability of reaching depths of 4,000 or 6,000 m depending on the model, are in operation. These floats measure temperature, conductivity, and depth with a precision of 0.001 °C, 0.002 PSU, and 3 dbar, respectively (71). The model capable of reaching 6,000 m, possesses a spherical design (Figure 3).

Since 2016, the Biogeochemical Argo program has been in operation, with the objective of increasing the number of parameters registered, including oxygen, nitrate, pH, chlorophyll-a, suspended particles, and downwelling irradiance. The primary rationale for this initiative was to facilitate the study of fundamental phenomena related to climate change, such as acidification and deoxygenation (61). According to the information provided by OceanOPS and the Biogeochemical Argo Network (72) there are today (March 2026) approximately 900 floats operational, all of which are equipped with an oxygen sensor, more than half with suspended particles, pH and nitrate sensors, and over 100 with an irradiance sensor as well. Based on these information, it is estimated that in March 2026, there were 130 floats fully equipped with all the BGC sensors. Since 2014, the deployment of more than 250 BGC-Argo floats has been facilitated by the Southern Ocean Carbon and Climate Observations and Modeling (SOCCOM) program (73).

Following an operational life of between 3 and 6 years, the lithium batteries of the floats expire and are unable to reach the surface. At this point, seawater begins to fill the buoy’s hull, initiating a corrosive process. Eventually, the buoy reaches the seafloor, where decomposition processes take place over the course of several years (74). Consequently, the environmental impact of the programs has been thoroughly examined (75), as there is no possibility of retrieval of the floats at the conclusion of their operational life. For example, one environmental impact arising from the operative life is the small amount of biocide, tributyl tin oxide (TBTO), released during the initial stage of each profile to prevent biological fouling of the conductivity sensors. The majority of the impact, however, occurs at the end of the float life, and thus, considerable efforts are in place to extend the battery life, decrease energy consumption and extend the operating lifetime, with troubleshooting and problem solving facilitated by the two-way communication provided by the Iridium network. According to the Euro-Argo information, there are currently more than 16,000 inactive floats lying on the seafloor, covering an area equivalent to just two football fields. However, these floats are separated by approximately 300 km, resulting in the dilution of a limited number of chemical components into the ocean. Each of these floats is composed of 8% plastic, 70% aluminum, and 22% components of a lithium battery, including 70% lead, 4% zinc, 9% copper, and 17% lithium. The amount of chemical components released into the ocean by Argo floats is equivalent to 90 kg of copper, 17,000 kg of aluminum and 180 kg of lithium (74). To contextualize this picture, the study by Morris and colleagues (70), published in 2024, asserts that it would require over 176,000 years of Argo operations to introduce a quantity of aluminum into the ocean equivalent to the annual global consumption. To illustrate this point further, it is noteworthy that the human contribution of plastic to the ocean, as represented by a single year of Argo floats, is equivalent to 4.4 million years of plastic input from these devices. Similarly, the natural flux of lead into the ocean is equivalent to 83 million years of Argo operations. It is important to note that any TBTO remaining in the float after it sinks to the seafloor will likely end up in marine sediments, where it will break down into inert, harmless components within a few weeks (75). Finally, it must be considered that locating and recovering each float at thousands of meters deep, would require extensive vessel deployment across ocean basins and systematic seafloor mapping. Such an approach would entail considerable technical challenges and potential environmental impacts.

#### 3.2.2 Moorings

Moorings are defined as anchored devices suspended in the water column, consisting in some cases, of a surface float that measures variables in the surface/subsurface and overlying atmosphere, and a cable or nylon rope that connects the surface float and the seafloor (60). This segment of the instrument can be equipped with additional sensors to measure parameters at precise depths. Mooring is fundamental to the study and monitoring of parameters over time in a specific and interesting location, and buoys can be classified based on their purpose and the set of sensors installed. Examples of moorings include physical oceanographic moorings, meteorological moorings (which also consider sea surface temperature and sea state), wave moorings and ecosystem moorings, which also measure biogeochemical parameters to monitor, for example, harmful algal blooms (HABs) (76).

Several networks around the globe control hundreds of buoys, coordinated internationally by Data Buoy Cooperation Panel (DBCP): in the US efforts are coordinated by the National Oceanic and Atmospheric Administration (NOAA) National Data Buoy Center (NDBC), the Canada’s Weather Buoy Network consist of 40 meteorological mooring, and analogous networks are in operation in Australia, the UK, India, Ireland, Spain, Korea and numerous other countries (76). Observation from NOAA and other international partner buoys can be visualized in real time at the NDBC website (77). Mooring can be classified according to their specific program and purpose: in NOAA network includes programs as Deep-ocean Assessment and Reporting of Tsunamis (DART) (78) and the Tropical Atmosphere/Ocean (TAO) (79) (Figures 2c,d) both in place since 1990s. The primary objective of the DART network is to facilitate the early detection of tsunamis in regions where such events have a history of occurring, with a particular focus on the Pacific coastline, which encompasses a total of 39 stations. Each station is composed of two components: an anchored seafloor Bottom Pressure Recorder (BPR) and a companion moored buoy. The BPR is responsible for the collection of real-time observations, while the buoy serves as a communication platform, facilitating an acoustic link that transmits the BPR’s data to the Iridium satellite system. The BPRs measure temperature and pressure at 15-s intervals, with the pressure, corrected for temperature, being converted to estimate sea-surface height (78). TAO is a buoy network comprising approximately 70 moorings sited in the Tropical Pacific Ocean to monitor fundamental currents as El Niño. These moorings consist of floating 2,3 m toroid buoys, equipped with antennas and meteorological instruments, which measure sea surface and conductivity at 1 m depth and at several intervals along the nylon rope that connects them to the seafloor. In certain locations, also current meters are installed with subsurface current mooring to measure pressure and temperature (79). Other relevant programs, among other, that use moorings are The Hawaii Ocean Time-series (HOT) program, Bermuda Atlantic Time-series Study (BAST) and the Ocean Observatories Initiatives (OOI). HOTS is a long-term oceanographic study based at the University of Hawaii at Manoa, with at a deep-water oceanographic station north of Oahu, Hawaii since October 1988. The primary objective of the HOT program is to obtain high-quality time-series measurements of selected oceanographic properties, including water mass structure, dynamic height, currents, dissolved and particulate chemical constituents, biological processes and particulate matter fluxes (80, 81). BAST is the world’s longest-running time-series for physical oceanographic data, including temperature, salinity, and dissolved oxygen measurements, with scientists visiting the station biweekly since 1954 (82). OOI is a network of 5 arrays where both fixed and mobile platforms including surface buoys, and inductive mooring cables, are deployed. The OOI manages and integrates observations from more than 900 instruments deployed on its five arrays, collecting from physical to geological and biological parameters (83).

**Figure 5:**
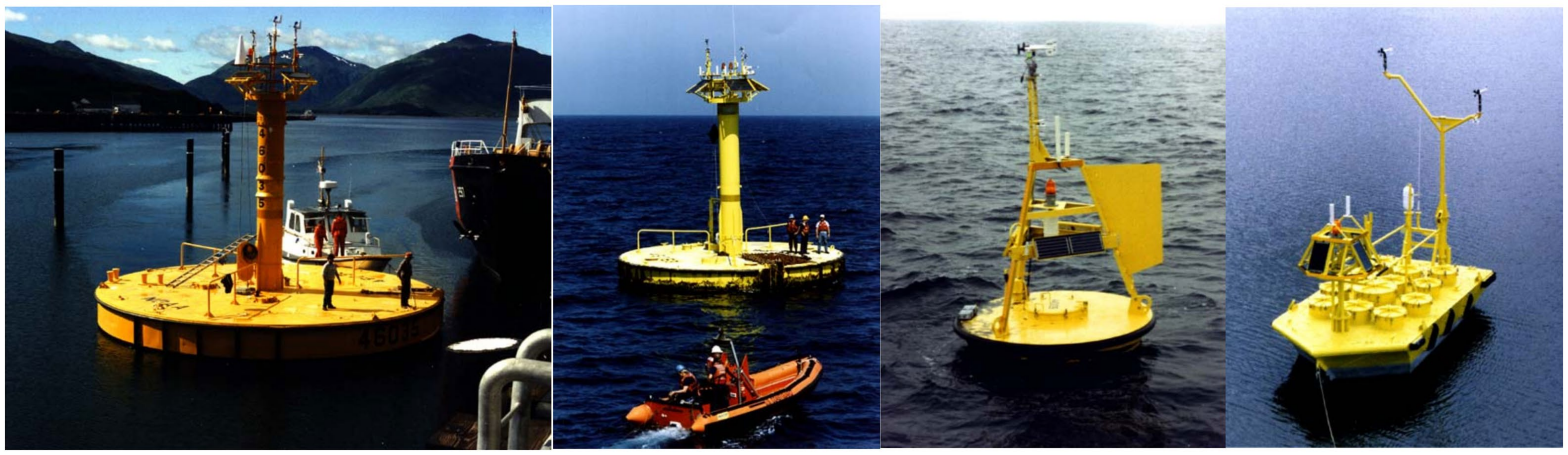
NDBC moored buoys with (from left to right) 12, 10, and 3 m. On the right 6 m shaped buoy all from https://www.ndbc.noaa.gov/faq/hull.shtml.

The dimensions of moorings can vary, with the NDBC utilizing buoys measuring 3, 10, and 12 meters in diameter, in addition to 6-metre boat-shaped buoys (NOMAD) (Figure 5), depending on the deployment location (costal or offshore) and the measurement requirements (84). There are numerous documented instances of large moorings worldwide, including the ODAS Italia 1, a buoy anchored in the Ligurian Sea, that has collected seawater and atmospheric parameters since 1970s (85). The buoy is a 41-metre-long steel pole divided into four sections, of which the uppermost three are watertight, while the fourth, submerged section is filled with water at a depth of six meters below sea level. This submerged section contains a small laboratory capable of accommodating two people. The buoy can record a wide range of parameters, including meteorological parameters, as well as measurements of sea temperature, salinity, turbidity, chlorophyll, dissolved oxygen, pO_2_ and nutrients. In Italy, there are other similar structures in the Adriatic Sea, near Venice and Trieste, and in the Mediterranean Sea, near Lampedusa (86).

### 3.3 AniBOS: animals with sensors

**Figure 6:**
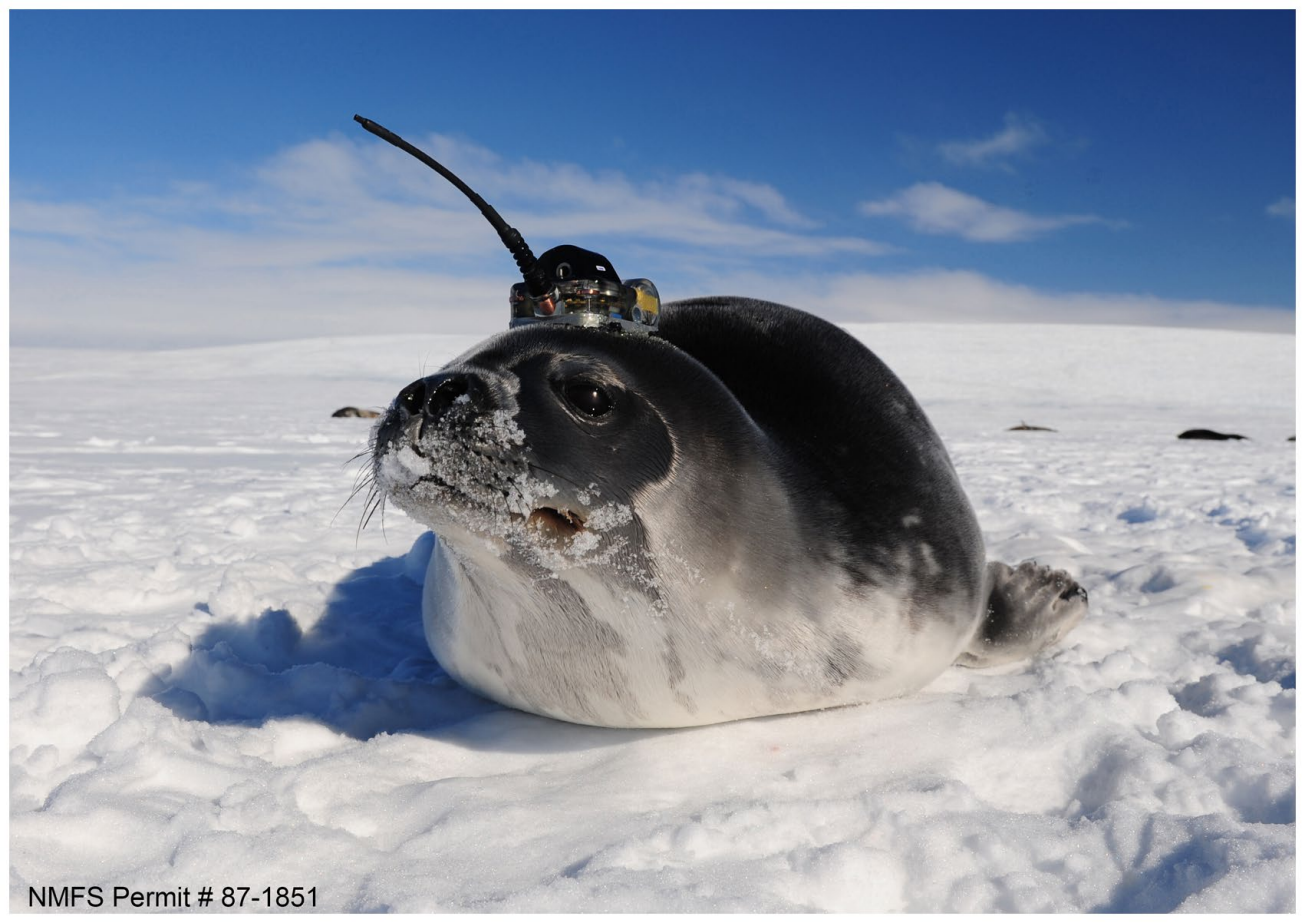
Weddell seal in the Ross Sea region carrying a CTD tag. Photo by Dan Costa taken under NMFS permit #87–1851 (from https://www.meop.net/pictures/dan-costa.html).

The enhancement of satellite networks and the development of ice-avoidance algorithms have rendered the Arctic and Antarctic Oceans more accessible for profiling floats to record observations about high-latitude oceans, which are undergoing rapid changes and are particularly susceptible to the impacts of climate change. However, the necessity for additional *in situ* observations in these regions has been identified, leading to the establishment of another network as part of the GOOS, known as the Animal Borne Ocean Sensors (AniBOS). Electronic tags are attached to marine animals and are equipped with integrated miniaturized temperature and conductivity sensors, enabling the collection of data pertaining to both the surface and the water column. Additionally, the tags provide information regarding the animal’s behavior in relation to the in situ environmental conditions (18). Animals can also carry biogeochemical sensors for chlorophyll and dissolved oxygen, thus improving the information provided by the BGC-Argo network. Compared to other programs, AniBOS starts more recently, in 2002 and was officially recognized by GOOS in 2020 (18): As of 2021, 1,480 tags had been deployed on animals (18) and the GOOS report 2025 reports 35 elephant seals tagged from December 2024 to February 2025 (24). Animals are quite useful because inhabit many of the poorly sampled regions, swim for multiple times per day also at several hundred meters of depth. Furthermore, sensors can be carried by a wide range of marine vertebrates, including marine mammals (seals primarily), seabirds, turtles, sharks, and other fish. The choice depends on the desire depth which is dependent on the characteristics of the animal in question and the capabilities of the capture facility. For instance, phocid seals can be easily captured when out of water, devices can be affixed to the animal’s fur, head, or back, causing minimal discomfort (Figure 6). Cetaceans (narwhals, beluga and bowhead whales) have been employed, taking advance of their migratory behavior. Although they are not the most optimal platforms overall, they can offer advantages over pinnipeds, as their fully aquatic and hairless bodies facilitate certain aspects of instrument deployment (18). Other animals include seabirds (king and emperor penguins), particularly for observations collected at depths of up to 100 m, and flying seabirds for surface measurements of ocean currents and winds (18). The network has declared its commitment to animal welfare and best animal handling practices on its website (17) and an ethical advisory board has been established. Furthermore, numerous publications in the last decade have outlined best practices for device use on board animals (87, 88). The sensors must be particularly miniaturized and able to accumulate data and communicate it to the Argos satellite network when the animal reaches the surface, given that marine mammals spend a very brief amount of time at the surface and so transmission opportunities are brief and infrequent, or generally not controllable, as in the case of the profiling floats. The primary sensors installed on animals are pressure, temperature and conductivity (from which salinity and density of seawater are derived) sensors, which record parameters with a high degree of precision (0.005 °C and 0.005 mS/cm) respectively. In addition other parameters are currently measured including fluorescence to estimate chlorophyll-a, dissolved oxygen and acoustic. Accelerometers and magnetometer are used to estimate wind speed, sea state and wind direction (18). or to study animals’ behavior as reported by Siegelman and coworkers (89).

**Figure 7:**
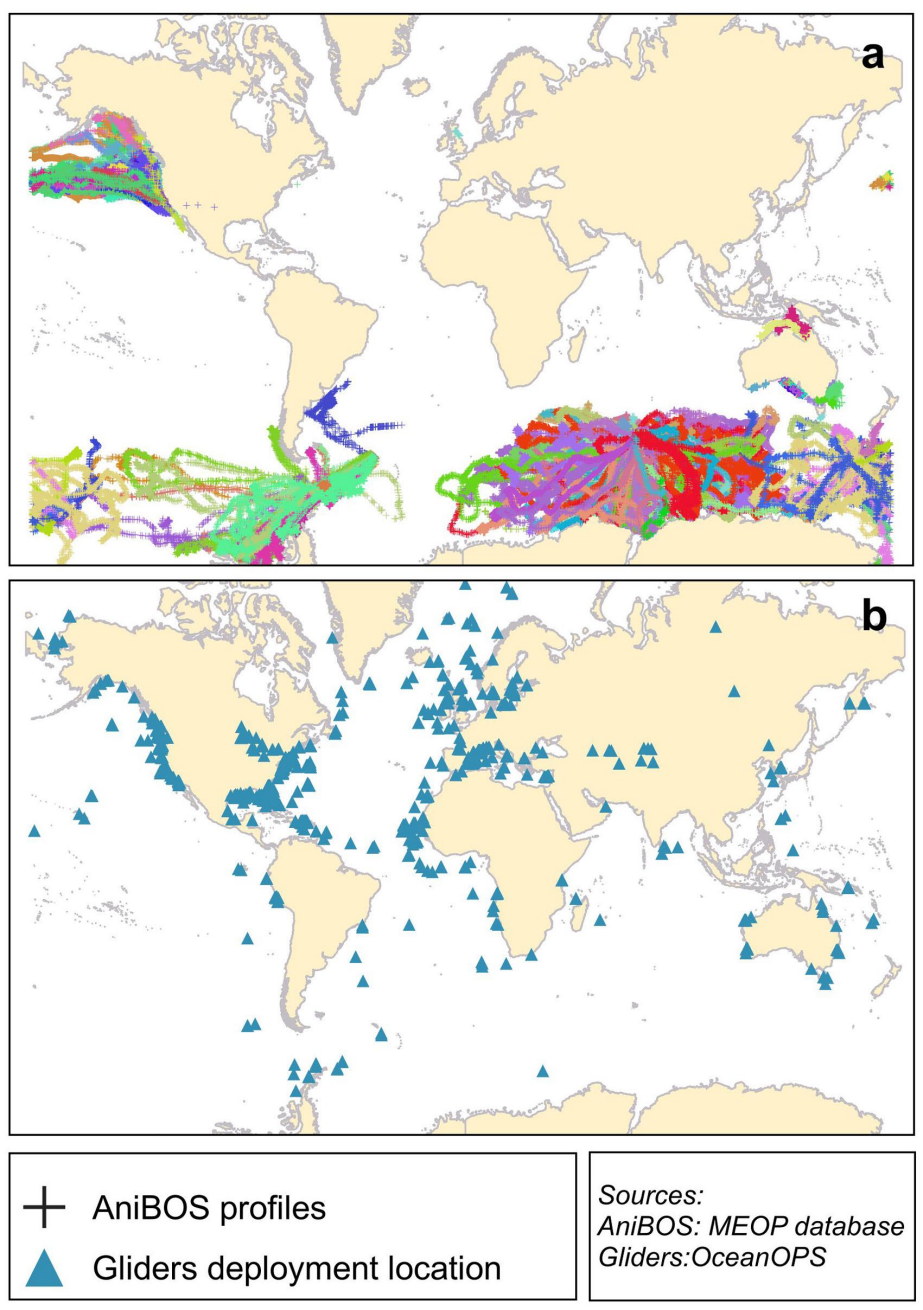
**(a)** Tracks of animal wearing sensors (AniBOS). Each color represents a different tracks **(b)** Gliders deployment locations. AniBOS data are download from https://www.meop.net/database/, gliders downloaded from OceanOPS (https://www.ocean-ops.org/board#).

As illustrated in Figure 7a the majority of animals are utilized in the Southern Ocean, and since the commencement of the project, more than 800,000 profiles have been collected (90).

### 3.4 Autonomous ocean observation platforms: gliding robots

Autonomous Ocean Observation Platforms (AOOP) are vehicles capable of traversing oceans, with minimum intervention necessary. In contrast to profiling floats such as Argo, these platforms possess the capacity to move continuously up and down and horizontally, gliding through the water and acquiring parameters, contingent on the sensors installed. Since 2016 *OceanGliders* have been incorporated into the GOOS, yet even prior to this, they constituted a pivotal element of the ocean observation system, particularly in the context of acquiring fine-spatial-resolution observations in regions proximate to ocean boundaries, thereby bridging the gap between coastal and open-ocean regions (Figure 7b). The integration of gliders has the potential to transform regional oceanographic observation, akin to the transformative impact of Argo in the domain of open-ocean observation (21). Gliders can fill the gap in regions where Argo floats do not have coverage or will rapidly drift away (e.g., continental shelf or boundary currents) and in situations where higher sampling frequency is required, because an Argo float complete cycle is 10 days while a glider can collect and transmit data in just few hours (91).

**Figure 8:**
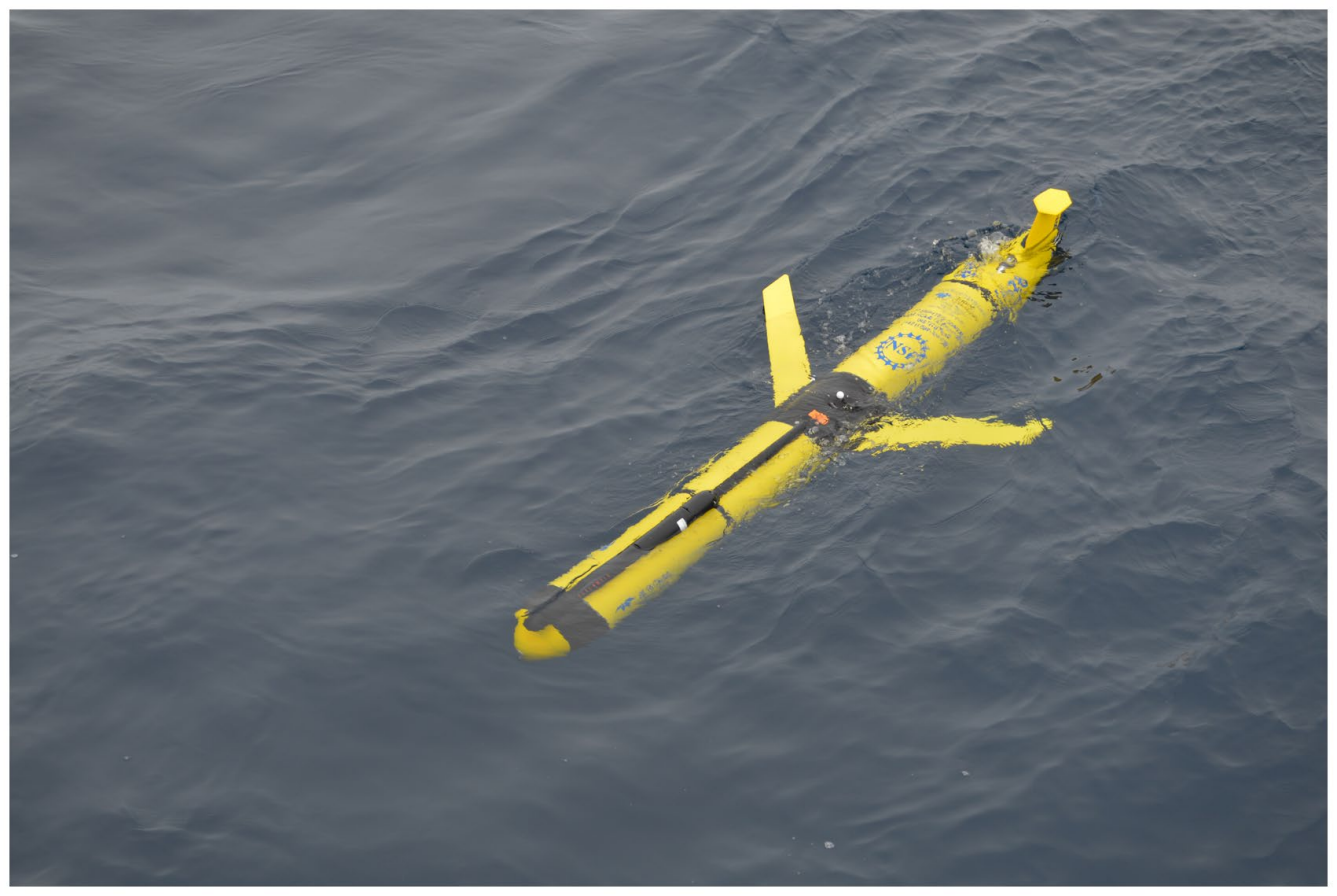
Underwater glider. Photo credit: Sheri N. White from https://oceanobservatories.org/image-gallery/.

The AOOP family encompasses a variety of vehicles, including manned submersibles, AUVs (autonomous underwater vehicles), ROVs (remotely operated vehicles), underwater gliders, and wave gliders. Each of these vehicles possesses distinct characteristics, such as operational time, operational range, measured water, and the level of autonomy. Manned submersibles and ROVs necessitate a high level of human control, either directly inside the vehicle or remotely. Conversely, AUVs and gliders are entirely autonomous, and the latter are also low-cost compared to the other platforms, thus being the most prevalent (92). There are three primary categories of gliding robots: underwater gliders, wave gliders, and multifunction gliders, which are still in conceptual design. The primary distinction among these categories lies in their structural design, with underwater gliders exhibiting a monomer torpedo-shaped configuration (see Figure 8). These gliders can be equipped with either wings and a propeller propulsion system or be configured without such features. In contrast, wave gliders and multifunctional gliders are characterized by their multi-body structures. A further distinction pertains to the power supply, with underwater gliders offering the option of electric, solar or thermal energy propulsion. Conversely, wave gliders are exclusively dependent on wave energy and solar energy for their propulsion (92).

Underwater gliders are capable of vertical movement by altering their buoyancy and horizontal movement by utilizing wings. These devices are equipped with advanced navigation, positioning, propulsion, and control systems (93) enabling them to operate autonomously or receive diving instructions from a monitoring center via wireless or satellite communication (typically the Iridium network) through the antenna mounted on the top of the vehicle. The driving of the vehicle is achieved by the forward movement of the internal mass (typically the battery), which in turn results in the forward movement of the center of gravity, causing the bow to descend and the tail to ascend. Concurrently, the oil pump functions to alter the vehicle’s buoyancy by pumping oil into a bladder, thus enabling the glider to dive into the water. The range of depths attainable by different models varies, with some capable of descending to depths of up to 6,000 m, and a glider manufactured by Tianjin University in 2018 achieving a depth of 8,213 m (92). The operational longevity of the glider is contingent upon the capacity of the lithium-ion battery, which typically spans a few months. To enhance glider endurance and facilitate the successful completion of long-term missions, solar-powered underwater gliders have been developed. In situations where the battery is in a low state, gliders are programmed to cease diving and transition to charging on the water’s surface. The management of battery life entails several considerations, including: (a) the frequency of pump usage, (b) the duration of pump usage, (c) the glider’s shape and the number of sensors installed that modify aerodynamics and water resistance, and (d) surface communication: frequency and time of satellite transmission, which could be every 3–6 h for surface missions or 8–11 h for deep missions (91). Those vehicles are design to spent only a few months in the ocean, in contrast to Argo floats, they must be delivered, recovered, and thus necessitate additional logistics and cost considerations, however, they can be used multiple times and do not become a waste at the end of their operational life. The delivery can be performed with both small and large vessels, depending on the distance from the coast and with due consideration for weather conditions (wind < 10 km/h, waves <1 m) to avoid damaging instrumentation. On board, underwater gliders can carry every type of sensor (e.g., temperature, pH; dissolved oxygen, chlorophyll, turbidity, nitrates, pCO_2_) but in many cases they should be designed especially for gliders (91).

### 3.5 Ships of opportunity and research vessels: introduction of new variables

As previously mentioned, the systems described in the preceding sections can measure some of the EOVs; however, as discussed in section 2, other variables must be considered. These include concentration of POPs, heavy metals and microplastics, as well as microbiological facies, ARGs and viruses. These parameters are characterized by high spatial and temporal heterogeneity, strong dependence on local sources and transport processes, and, particularly for microbiological components, rapid variability driven by environmental conditions such as temperature, nutrient availability and hydrodynamics. The investigation of these parameters is less extensive compared to that of the aforementioned ones, and sampling activity is often required in remote offshore areas, which can only be reached by vessel. The inability of satellite-based monitoring to detect or quantify these variables naturally precludes the possibility of achieving simultaneous global coverage. However, the international community aims to collect as much data as possible, including construction time series, in order to study the evolution of emission phenomena in response to the introduction of new restrictions on molecules deemed to be hazardous to human and environmental health. In this context, the harmonization of sampling protocols and analytical methodologies is essential to ensure data interoperability and long-term usability. In addition to sampling issues, a further challenge pertains to the laboratory analysis of these samples. This process is often lengthy and complex, necessitating the expertise of dedicated personnel, the pretreatment of samples, and a significant time investment before results can be obtained.

**Figure 9:**
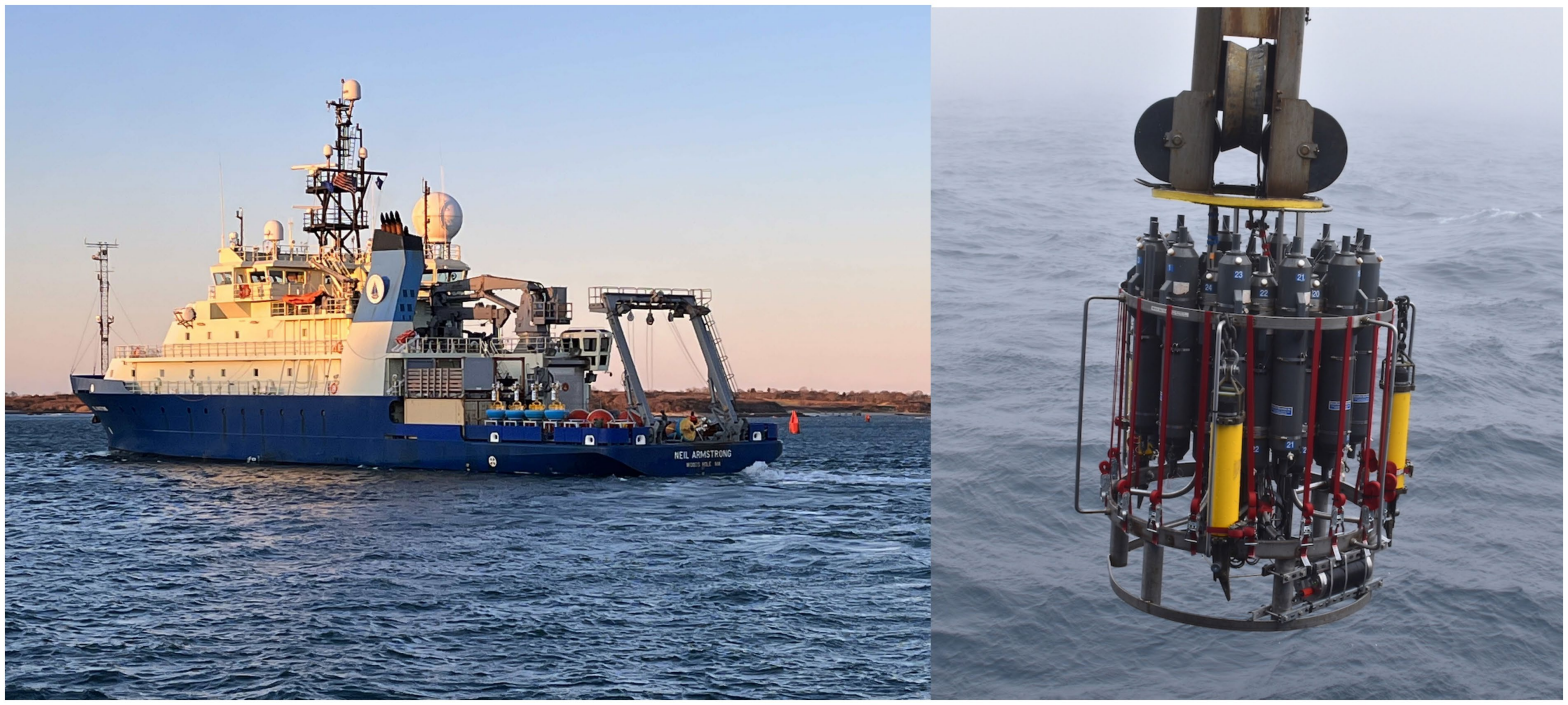
Left: The R/V Neil Armstrong left the dock at Woods Hole, credit Dee Emrich © WHO; Right: Rosette deployment, credit L Sawyer Newman © WHOI, both from https://oceanobservatories.org/image-gallery/.

During the initial centuries of oceanic exploration, ships constituted the sole means of observing the open ocean (26). Also today, with satellites that measure the surface and a limited number of autonomous devices capable of reach deep ocean, research vessels (Figure 9) remain indispensable for deep and open ocean sampling. These vessels are meticulously designed for specific purposes and are indispensable for projects such as GEOTRACES (94) which monitor the concentration of trace elements, micronutrients and isotopes around the globe. Research vessels play a pivotal role in the deployment of surface drifters, profiling floats, mooring and gliders. The collection of deep seawater is facilitated by rosette systems (Figure 9) which utilize Niskin bottles equipped with a CTD device. Research ships are also equipped with cranes for the deployment and retrieval of instruments and can be configured with specialized equipment for target missions. The principal disadvantages are the limited number of research vessel available worldwide, the requirement for specialized personnel and the high cost.

Over the years, a new approach has been implemented, the Ships Of Opportunity (SOO), vessels such as ferryboats or a cargo ship, that using autonomous devices are capable of collecting seawater and registering fundamental parameters without interfering with the ship’s main mission. The use of ships for scientific purposes is a long-standing practice. For centuries, the temperature of water and meteorological parameters were recorded in ships’ logbooks (95). The first international standard for observation practices was set at the 1853 Brussels Conference (96). Since 1931, the Continuous Plankton Recorder at Plymouth Marine Laboratory is in place, the longest running, most geographically extensive marine ecological survey in the world. Since the first Continuous Plankton Recorder was towed by the SS Albatross between Hull, England and Bremen, Germany, more than 300 vessels have helped in a voluntary capacity to maintain the Survey, contributing building a considerable database of marine plankton (97). Another valuable example is the Oleander Project that since 1977, equips the MV Oleander, owned by the Bermuda Container Line, with equipment to measure temperature, salinity and CO_2_. All these parameters where measures from the continental shelf near New Jersey, to the Slope Sea, the Gulf Stream, and part of the Sargasso Sea near Bermuda (98).

**Figure 10:**
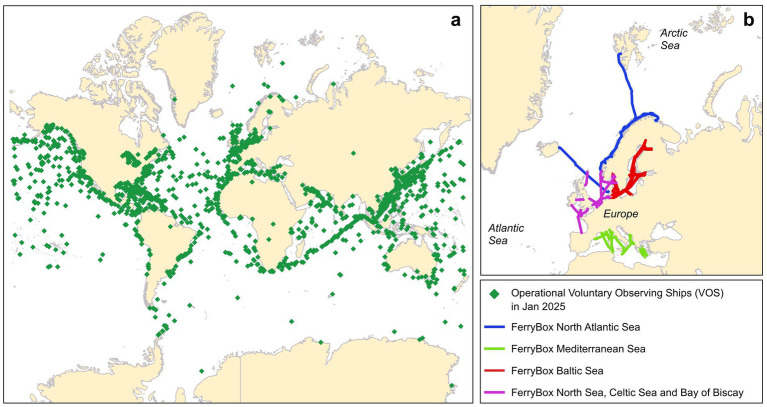
**(a)** Operational Voluntary Observing Ships (VOS) transmitted location in January 2025, data downloaded from OceanOPS (https://www.ocean-ops.org/board#). **(B)** FerryBox route in North Atlantic, Baltic, and Mediterranean Sea downloaded from https://www.ferrybox.org/routes_data/routes/index.php.en. Data from North Sea where not downloadable and were reconstructed from the XLS file available at https://www.ferrybox.org/routes_data/routes/table_of_routes/index.php.en, updated in 2019.

More recently, the SOO scenario has been organized with the Ship Observation Team (SOT), inserted inside the GOOS, that has different programs inside: the Voluntary Observing Ships (VOS) (99) and the Ship Of Opportunity Program (SOOP) (100). VOS coordinates the monitoring of marine meteorological parameters by around 2,000 cargo ships and other types of vessels around the world (Figure 10a). The ship crew or automated weather stations measure variables at the sea surface (e.g., temperature, waves, currents and ice) and variables related to the atmosphere above the sea surface and all information are transmitted from ship to shore via satellite. SOOP is dedicated to the collection of data pertaining to eXpendable Bathy Thermographs (XBTs), encompassing the study of boundary currents, the absorption of carbon dioxide from the atmosphere (see for example the ongoing collections on NOAA portal (101)), biogeochemical parameters and temperature/salinity. XBTs are temperature probes that are launched from the bridge of a ship by the ship personnel 4 or 6 times a day or 20–30 times a day (High-Density XBTs Program). Temperature and salinity are recorded through ThermoSalinoGraphs (TSG), instruments, automatically operated, that measure salinity and sea surface temperature every 10 s, or approximately 100 m along the ship track. Ships of the SOOP are also used to deploy Argo floats or GDP drifters.

Another relevant SOO initiative was initiated from 2003 to 2005: a European-funded multinational collaboration known as FerryBox, with the objective of optimizing a system for automated measures and water sampling on board ferries navigating the European seas. One of the major advantages of ferries is that their route is constant and repeated at precise intervals along the seasons, thereby enabling the collection of time-series data for the study of parameter variations over the course of years. A total of 30 ships were selected for the study, representing several ferry lines (Figure 10b). In the North Atlantic the Norwegian Institute (NIVA) operated the project along the following routes from Bergen to Kirkenes, from Bergen to Hirtshals and from Oslo to Kiel. In the Baltic Sea operation were coordinated by the Finnish Institute of Marine Research between Helsinki and Stockholm, Helsinki-Tallin and Tallin-Stockholm. In the North Sea operations were overseen by the Helmholtz–Zentrum Geesthacht (Germany) encompassing routes connecting the United Kingdom, Belgium, the Netherlands, Germany and Norway. Projects were also initiated in the Atlantic, coordinated by the French institution IFREMER, in the Western Channel and the Bay of Biscay, and in the Mediterranean Sea between Italy and Greece (96). A specific apparatus, also called FerryBox following the namesake EU project, was installed in all the ferries involved in the program. This apparatus consists of a water inlet through which water is pumped into a measuring circuit containing multiple sensors and a sampling system. The parameters recorded depend on the sensors installed, and these parameters may include temperature, salinity, turbidity, chlorophyll-a, oxygen, pH, pCO_2_, algae sensors, as well as meteorological parameters. The operational control of the FerryBox is facilitated by a computer system that logs the data and facilitates its transmission to the shore via mobile phone or satellite communication (96). Following the initial collaborative development in the early 2000s, specific projects employed the FerryBox system, including microplastics monitoring along the Oslo-Kiel route in 2019 (102). In this study, seawater (5,000–15,000 L) was automatically collected at a depth of 3 m on the starboard side of the ferry and was passed over a series of metal sieves to collect three different particles dimension (100, 300 and 500 μm). The FerryBox system has also been employed in the detection of contaminants of emerging concern and pesticides, as reported in two publications by Brumovsky and co-workers in 2016 (103) and 2024 (104), respectively. The first investigation was conducted in the North Sea around Germany, UK and Denmark, while the second in the northern part of the North Sea along the Norwegian coast, till the Svalbard Island and in the Baltic Sea. The sampling system consists of an inline automated sampler with a capacity of 24 one-liter bottles and the water sample collection events can be pre-programmed or triggered in real time remotely using an internet connection. With appropriate fluorescence sensors also oil spills can be detected on board ferries in a FerryBox system as reported by Part and co-workers who investigated the area between Estonia and Sweden and published their results in 2021 (105). Traditional FerryBox have a wardrobe footprint, but also pocket version, with just sensors, where developed and applied outside Europe, as for example in Brazil (106). Not only chemicals, but also marine microbial biodiversity can be investigated with ferries as reported in 2015 by Stern and co-workers (107) who used a sampling machine capable of automatically collecting seawater samples with a peristaltic pump and storing them in several plastic bags.

A notable limitation of ferries pertains to the spatial extent of the sea area that can be sampled, given their primary function of transporting passengers between proximate cities. Another application of the SOO concept involves the utilization of cargo ships that traverse vast maritime distances, establishing connections between continents and enabling the collection of data across extensive transects. In the past several projects were implemented as for example the Global Ocean Surface Underway Data (GOSUD) Project, established in 2001 as an initiative of the International Oceanographic Data and Information Exchange (IODE) to collect observations about temperature and salinity with research vessels, merchant ships, sailing ships or cruise vessels. This and other projects are described in recent review by Rosa and co-workers (108). A more recent application is described by Macdonald and co-workers (109), who tested an agreement with a shipowner in the Gulf of Mexico and described a new project, Science Research on Commercial Ships (Science RoCS) which aims to enhance research opportunities by equipping commercial vessels with suites of maritime-appropriate scientific sensors that operate autonomously on regular ship routes with minimal crew intervention. In addition to cargo ships, the potential extends to fishing vessels, as evidenced by Santos et al. in 2024 (110), who presented a prototype of a shipping board automated meteorological and oceanographic system (SAMOS), incorporating a Ferrybox, which was installed on a Portuguese research vessel to measure parameters such as temperature, salinity, pH, and numerous others (110). The primary constraints imposed by SOO pertain to the spatial coverage, which is particularly constrained for ferries and cargo, and is limited to the routes of the vessels, additionally measures are mainly at surface or at few meters below, with the exception of instruments as ADCPs (Acoustic Doppler Current Profiler) that measure currents through sound. A significant challenge is that this activity depends on project funding, and it often concludes when the funds are exhausted. Vessel operators and owners are not expected to contribute financially to the maintenance of the system (108). In addition to passenger ships like ferries, cargo ships, and fishing boats, collaborating with naval vessels is another effective strategy. The Sea Care Project, stemming from an agreement between the Italian National Institute of Health and the Italian Navy, exemplifies this replicable model for countries with naval forces (111). It goes beyond automated systems on cargo ships by allowing researchers to board non-research vessels. This enabled the collection of seawater samples for chemical analysis, microbiological and microplastic samples, plus EOVs measurements. The project is producing a global-scale study on human-induced marine contamination using consistent methods, yielding comparable results essential for ocean and human health perspectives.

## 4 Available databases

The oceanic data are dispersed, as outlined by Brett et al., and the *data tsunami* previously described has not been accompanied by a rethinking of how observations are collected, shared and accessed (7). They identified four issues (a) Silos: government agencies, companies and researchers collect data for their own specific purpose, (b) Control: there is often reluctance to share data with centralized repositories, (c) Format and quality: data are often non-interoperable and there is a problem of universal standard, (d) Fragmentation: there are multiple catalogues that overlap with each other. In addition to the issues identified by Brett et al., the authors of this review wish to emphasize the efforts and dedication of oceanographers in the collection and dissemination of current data.

Observations from satellites, buoys, animal sensors, and gliders are transmitted via satellite networks to shore-based processing centers, where they are processed and subsequently made publicly available. To facilitate data interpretation for all interested researchers — particularly public health and environmental researchers who may use these information to select optimal sampling locations, compare datasets, or explore correlations— this concise overview of available databases is provided for straightforward consultation and download. For example, microbiologists and environmental virologists may require temperature, salinity, dissolved oxygen and pH to study allochthonous bacteria or viruses founded in a specific location or may need nutrients concentration to investigate an HAB. Similarly, chemists conducting environmental water analysis may need to determine the direction of currents in order to select the most appropriate sampling location, and this would allow them to study the movement of POPs or microplastics, and to elaborate their results. In the context of satellite data, the utilization of the Copernicus Marine Service’s visualization tools^1^ is strongly advocated. These tools facilitate the visualization of EOVs at three distinct complexity levels. The MyOceanPro tool enables the download of data, contingent upon the selection of a specific time interval (e.g., daily or monthly average), a designated date range, a specified depth range, and a defined geographical area. The downloaded file are in NetCDF format and can be visualized and manipulated to create maps through a GIS software like QGIS^2^. In the event that profiling floats data are required, EuroArgo provides access to all Argo buoy observations, with the selection tool^3^ enabling the user to select network (Core, Deep and BGC), parameters, time interval and geographical area. Visualization of the information is possible online, with the option to select the desired buoy cycle, and download in NetCDF and CSV formats. All the *in situ* technologies described above are available to download from the Coriolis portal^4^. Additionally, there are tools specifically designed for visualization, such as Earth Null School^5^, which aggregates both oceanic and atmospheric information, and EMODnet^6^, which, regrettably, is limited to Europe, aggregates data from various sources, including EOVs, as well as other pertinent information for elaboration, such as the locations of potential anthropogenic pollution sources, including aquaculture implants, oil platforms, submarine volcanoes, desalination plants, and numerous others. A significant proportion of these data can be downloaded as shapefiles containing GPS coordinates. Another useful platform to have a complete scenario of all in situ available technologies is OceanOPS^7^, where all platforms can be visualized, GPS coordinates can be downloaded, and static maps can be generated for each month.

## 5 Discussion: new frontiers in ships of opportunity

As emphasized in Section 3.5, ships play a pivotal role in the collection of observation from the deep ocean and the procurement of water samples for chemical and microbiological analysis. However, it should be noted that if research vessels are not available, the utilization of SOO is recommended, with ferries, cargo, and fisheries vessels being optimal depending on the objectives of the investigation, the time interval, and the spatial and temporal parameters of analysis. A key challenge is that parameters, measurement, and sample collection must not disrupt vessel operations or their primary objectives, necessitating a high degree of automation. While the FerryBox system has been demonstrated to be effective for chemical parameter measurement, challenges may arise in the context of sampling collection for chemical and microbiological analysis, particularly in scenarios requiring high volumes, as evidenced in recent studies that detected traces of SARS-CoV-2 in oceanic waters (112) and examined the dissemination of antimicrobial resistance genes (45). A further challenge pertains to the intricate logistics of a cargo vessel, which is characterized by prolonged travel times, thereby giving rise to the issue of the optimal timing and method for the unloading of water samples collected for analysis. It is imperative that these samples are stored under stringent refrigeration or freezing conditions, a necessity that is particularly pronounced in the context of microbiological analysis.

In this discussion, the exploration of potential future solutions and novel frontiers to optimize the collection of data on board ships of opportunity is undertaken. The present dissertation is conducted with the objective of identifying the parameters deemed essential for measurement, the available technologies on the market that can be installed on board vessels, and the degree of adaptation, automation, maintenance requirements, advantages, and disadvantages of these technologies. The ensuing discussion will be structured around the findings presented in Tables 2, 3, which provide a concise summary of the key considerations. The tables are meticulously organized based on the degree of automation, with Table 2 detailing techniques that boast the highest degree of automation and Table 3 offering insights into those with an intermediate level of automation.

**Table 2:** Apparatus with high degree of automation.

**Type of apparatus**	**Parameter**	**Purpose of environmental and health analysis and**	**Adaptability**	**Degree of automation**	**Maintenance**	**Main characteristics**	**Limitations**
Sensors	Temperature, Salinity, Conductivity, pH, dissolved oxygen	Study of EOVs	High	High	Medium	Continuous monitoring and early warning	Periodic maintenance
	Turbidity	Study of EOVs	High	High	Medium		
	Chlorophyll-a	Study of EOVs	High	High	Medium		
	Oils and methane	Spills detection	High	High	Medium		
	CO_2,_ BOD, COD and TOC	Study of EOVs	High	High	Medium		
	Coliforms	Indicator parameters for fecal contamination of marine and marine coastal waters	High	High	Medium		
	Macro and micro nutrients and UV–VIS screening	Eutrophication and indirect HAB monitoring and presence of POPs	High	High	Medium		
	Blue-green algae	Direct monitoring of HABs	High	High	Medium		
Discrete analyzers	Nutrients, micronutrients, alkalinity, chloride and others	Eutrophication and indirect HAB monitoring	High	Medium	Medium		Periodic maintenance, reagent refill and discharge
portable NIR spectrometer-Chemometrics	Organic compounds, depending on the model	Fingerprint identification to define an anthropic contamination index	High	High	Medium	Analysis in few seconds and quick response	Periodic maintenance

BOD: Biochemical oxygen demand, COD: chemical oxygen demand, TOC: total organic carbon. Adaptability: High = possibility of installing on board small and large vessels, Medium = necessity of medium or large vessels. Degree of automation: High = support only in preparation stage, Medium = necessity of loading and manual recovery, Low = necessity of human support. Maintenance: Medium = calibration can be controlled remotely, Low = Manual calibration or other operations that requires human support.

**Table 3:** Apparatus with medium or low degree of automation.

**Type of apparatus**	**Parameter**	**Purpose of environmental and health analysis and**	**Adaptability**	**Degree of automation**	**Maintenance**	**Main characteristics**	**Limitations**
Extraction system	POPs	Research of presence and concentration of POPs, able to bioaccumulate and biomagnify along the trophic chain	Medium	Low	Medium	Volume reduction	Organic solvent disposal, not automatic samples loading nitrogen and, refrigeration required
Filtration system	Microplastics	Research of presence and concentration of microplastics, able to accumulate in tissues	High	Medium	Medium	Sampling of subsurface microplastics	No surface microplastics sampling, filter replacement required
	Virus and Bacteria	Genomic characterization of virus and bacteria: presence of allotones bacteria and ARGs.	High	Medium	Medium	Volume reduction	Filter replacement, disinfection and refrigeration required

Adaptability: High = possibility of installing on board small and large vessels, Medium = necessity of medium or large vessels. Degree of automation: High = support only in preparation stage, Medium = necessity of loading and manual recovery, Low = necessity of human support. Maintenance: Medium = calibration can be controlled remotely, Low = Manual calibration or other operations that requires human support.

The employment of sensors, an extensive array of instruments capable of measuring various parameters (see Table 2), is characterized by a maximum degree of automation and a high degree of adaptability. These sensors can be equipped with or without a reagent, the latter requiring periodic replacement. Despite this minimum degree of human intervention, the sensors can operate automatically, immersed in a tank where seawater is pumped during the cruise. The sensors demonstrate adaptability to diverse vessel types and possess the capacity for uninterrupted operation, with parameters being systematically registered and transmitted to shore via satellite connectivity. This functionality facilitates the utilization of the sensors as an early warning system, capable of disseminating critical information to both the vessel bridge and onshore locations. It is imperative to acknowledge that sensors necessitate a modicum of maintenance, encompassing periodic calibration and reagent replenishment.

With a reduced degree of automatization, summarized in Table 2, there are online discrete analyzers, in which different samples are loaded into reaction cuvettes in which a colorimetric reaction is performed to measure nutrients, micronutrients, alkalinity, chloride and other parameters. The reagents required are automatically transferred from a tray into the reaction cuvettes, which are then filled with seawater. The colorimetric reactions occur, and the parameters are measured using an optical sensor. In order to achieve full automation, it is necessary to consider two aspects: firstly, the cuvettes should be filled by another automatic system, and secondly, the start of the colorimetric reaction must be automatized, because instruments now available on the market are design to be managed by an operator through a PC and in fact the other downside is that reagents must be refill and discharged periodically.

The last fully automatic instruments describe in Table 2 is a handled NIR spectrometer, which operates in near infrared (wavelength 780–2,500 nm). When employed in conjunction with chemometrics, the portable NIR platform possesses the capability to identify and quantify a multitude of molecular types, contingent upon the construction of a chemometric model (113). The analysis of water by NIR is in its infancy, with the development of Aquaphotomics (114, 115) providing a new avenue for research. This field studies NIR water absorption and its applications in the analysis of substances dissolved, including aromatic hydrocarbons (116, 117) and non-polar organic compounds (118). Handled NIR instruments can operate in transmittance mode and can be immersed in a tank with other sensors. The most significant feature of portable spectrometer technology is its ability to generate a unique fingerprint of seawater, which can be compared with other samples.

The analysis of emerging contaminants at low concentration levels (generally ng/L) or specific virus or bacteria cannot be performed with sensors but requires high performance equipment that cannot be placed onboard vessels. The implementation of upgrades on board SOO is contingent upon the integration of systems that possess the capacity to enhance the methodologies employed in sampling procedures, pre-treatment and sample concentration. The objective of these enhancements is to circumvent the necessity of drawing substantial volumes of seawater, a prerequisite for conducting both chemical and microbiological analyses.

As discussed in section 3.5, FerryBox were utilized for sampling of microplastics, though such sampling presents significant challenges aboard vessels. Microplastics tend to float on the surface until losing buoyancy, requiring superficial sampling in undisturbed waters conditions difficult to achieve due to vessel sea intakes positioned several meters below the surface and ship motion disturbing the water, displacing floating particles (119). Notwithstanding these difficulties, sampling of not floating microplastics in the water column could be performed, collecting seawater from the vessel intake and making it through filters where particles could remain held (120). It is imperative to acknowledge the potential impact of adverse sea states on vessel positioning, which may result in variations in the depth at which sampling is conducted (121). Additionally, it is crucial to emphasize the necessity of replacing and storing filters after sampling.

In the context of chemical analysis, particularly regarding the quantification of POPs, standardized protocols typically necessitate the collection of several liters of seawater for analysis. This initial step is followed by a process of concentration and analyte extraction, which involves the use of small volumes of organic solvent to perform solid-phase extraction (SPE). This method ensures the retention of target analytes to the substrate, facilitating their subsequent elution and reduction of the original volume of seawater to a few milliliters (122). SPE can nowadays be performed with semi-automatic extractors where operators load the samples into the extraction column or membrane and a software controls solvents disbursement and all the extraction process. This type of equipment could be installed on board research vessels, however, the installation on board a SOO could be much more challenging. There are several issues to consider, including, but not limited to, the following: the disposal of organic solvents; the automatic loading of samples and extraction supports; the removal of tubes containing extracts; the need for nitrogen for the drying process of SPE media; and the need for refrigeration of extracts.

For microbiological analysis of virus and bacteria, filtration systems are required. For virologic analysis, hundreds of liters of seawater must go through a special electropositive filter capable of retain virus DNA/RNA the need for pretreatment (123). For bacteria analysis seawater is passed over tin sterile filters, with just a prefiltration need to remove sand and other components that could cause clogging. These systems are quite simple, require a pumping system and a jar or holder for the filter, the main challenges in the automatization process on board a SOO could be filter replacement after sampling, filers storage that must be frozen till analysis and disinfection procedures after each sampling.

## 6 Conclusion

This narrative review emphasize the pivotal role of ocean observation in enhancing our comprehension of the intricate interconnections between ocean and human health, It is designed to contribute to the prevailing trend of expanding EOVs. In this context, it is recommended that new variables, relevant to OHH, including POPs, AMR, and others, be measured in the ocean environment.

To properly contextualize these emerging variables, several key oceanographic data collection technologies were examined in detail, including satellites, buoys, drifting floats, moored floats, moorings, animal-mounted sensors, gliders, research vessels, and SOOs. For these methodologies and applications, the main characteristics and limitations were also systematically summarized, with the aim of improving their relevance by explicitly incorporating a public health perspective. In this context, particular attention was given to how each platform can contribute to the monitoring of environmental conditions that influence human health and climate-related stressors, thereby underscoring their role not only in the acquisition of ocean data, but also in supporting the interpretation of new variables of interest within an integrated and applied framework.

In parallel, the review has summarized the main public repositories currently available for accessing ocean observations, underlining the importance of data availability, interoperability, and accessibility for both scientific research and policy and management decision.

The authors’ proposed perspective aligns with the United Nations Sustainable Development Goals (SDGs), particularly SDG 3, SDG 13, and SDG 14. In particular, SDG 3, which aims to ensure good health and well-being for all, cannot be fully achieved without also considering the other two. Together, these goals underscore the need to strengthen ocean observation systems, enhance environmental monitoring, and foster a more integrated understanding of the links between climate change, the integrity of marine ecosystems, and human well-being.

Looking ahead, greater integration between the disciplines of oceanography and public health is desirable to support a transdisciplinary approach. Shared protocols, harmonized metadata, and increased data exchange would enhance the usefulness of observations and strengthen their policy relevance. This viewpoint is in line with SDG 17, which highlights the significance of partnerships in achieving sustainable development. In the context of ocean and human health, SDG 17 promotes stronger collaboration among researchers, public institutions, policymakers, and stakeholders to achieve standardization of data-sharing practices and observational strategies.

Finally, the scalable use of Ships of Opportunity (SOO) holds transformative potential for EOVs data acquisition. SOO, already proven for existing variables such as pH, phytoplankton biomass and many others, could be adapted for monitoring the proposed variables, which would be cost-effective without dedicated research fleets, especially when research vessels are not available. This approach would extend data collection to less-observed areas and support both established and proposed EOVs.

## Footnotes

1 Accessible via https://marine.copernicus.eu/access-data/ocean​-​visualisation-tools

2
https://qgis.org/project/overview/

3
https://dataselection.euro-argo.eu/

4
https://www.coriolis.eu.org/Data-Products/Data-selection

5
https://earth.nullschool.net/about.html

6
https://emodnet.ec.europa.eu/geoviewer/

7
https://www.ocean-ops.org/board
